# Metabolic inputs in the probiotic bacterium *Lacticaseibacillus rhamnosus* contribute to cell-wall remodeling and increased fitness

**DOI:** 10.1038/s41522-023-00431-2

**Published:** 2023-09-26

**Authors:** Ronit Suissa, Tsviya Olender, Sergey Malitsky, Ofra Golani, Sondra Turjeman, Omry Koren, Michael M. Meijler, Ilana Kolodkin-Gal

**Affiliations:** 1 Department of Chemistry, Ben-Gurion University of the Negev, Be’er Sheva, Israel; 2 Department of Molecular Genetics, Weizmann Institute of Science, Rehovot, Israel; 3 Life Science Core Facilities, Weizmann Institute of Science, Rehovot, Israel; 4 Azrieli Faculty of Medicine, Bar-Ilan University, Safed, Israel; 5 Department of Plant Pathology and Microbiology, Faculty of Agriculture, Food and Environment, The Hebrew University of Jerusalem, Rehovot, Israel; 6 Present Address: The Scojen Institute for Synthetic Biology, Reichman University, Herzliya, Israel

**Keywords:** Microbiome, Bacteriology

## Abstract

*Lacticaseibacillus rhamnosus GG* (LGG) is a Gram-positive beneficial bacterium that resides in the human intestinal tract and belongs to the family of lactic acid bacteria (LAB). This bacterium is a widely used probiotic and was suggested to provide numerous benefits for human health. However, as in most LAB strains, the molecular mechanisms that mediate the competitiveness of probiotics under different diets remain unknown. Fermentation is a fundamental process in LAB, allowing the oxidation of simple carbohydrates (e.g., glucose, mannose) for energy production under oxygen limitation, as in the human gut. Our results indicate that fermentation reshapes the metabolome, volatilome, and proteome architecture of LGG. Furthermore, fermentation alters cell envelope remodeling and peptidoglycan biosynthesis, which leads to altered cell wall thickness, aggregation properties, and cell wall composition. In addition, fermentable sugars induced the secretion of known and novel metabolites and proteins targeting the enteric pathogens *Enterococcus faecalis* and *Salmonella enterica* Serovar Typhimurium. Overall, our results link simple carbohydrates with cell wall remodeling, aggregation to host tissues, and biofilm formation in probiotic strains and connect them with the production of broad-spectrum antimicrobial effectors.

## Introduction

Probiotic strains are defined as strains that confer health benefits to their consumers and can be consumed as fresh fermentation products or as dried bacterial supplements^1,2^. Lactobacillaceae and Bifidobacteria, are widely recognized as the most frequently used genera probiotics^1,2^. This industry heavily relies on strains initially isolated from humans or fermented products^3–5^. This industry is growing exponentially, but the most consumed products fail to meet the criteria for biotherapeutics. One overarching hypothesis is that uncovering mechanisms that contribute to the competitiveness of probiotic strains and their response to different diet components may improve the compatibility and predictability of these strains as live therapeutic agents^6^.

Lactobacillaceae are Gram-positive rod-shaped, facultative anaerobes that belong to the lactic acid bacteria (LAB) group. For LAB, the main end-product of carbohydrate metabolism is lactic acid^7,8^. Members of this family are core members of the microbiota residing in the gastrointestinal tract (GIT) of humans and animals^9^. Lactobacilli are widely used as probiotics and are frequently applied as a part of fermented foods and food supplements. The performance of Lactobacillaceae, as well as the performance of other probiotic strains in the gut, is heavily influenced by nutrient composition and availability in this habitat^10^.

Efficient fermentation plays a fundamental role in LAB physiology as these bacteria rely mainly on this core pathway to produce energy under anaerobic conditions or oxygen limitation. During fermentation, organic acids, alcohol, and carbon dioxide are generated via the oxidation of carbohydrates^11^. While it was generally believed that fermentation across LAB strains occurs uniformly in direct correlation with the capacity to utilize glucose^2^, we recently found the repertoire of the response of LAB strains to glucose varies and that the overall growth of the culture also reflects complex adaptation to this carbohydrate^12^.

One general feature significantly affected by fermentation in tested LAB strains was that glucose utilization shaped the colony morphology under static conditions, as all species exhibited morphological colony changes upon glucose treatment^12^. Specifically, the proficient probiotic bacterium *Lacticaseibacillus rhamnosus GG* (LGG) demonstrated the clearest alterations in cell shape and colony morphology that did not directly reflect cell growth^12^.

Here we utilized LGG as our lens to unravel the molecular mechanisms that act downstream to glucose metabolism and contribute to altered fitness upon glucose utilization. We used an unbiased approach, mapping the overall changes in the proteome, metabolome, and volatilome of LGG in the presence and absence of fermented sugars. We aimed to determine the extent to which this model probiotic strain alters features that could contribute to the success of probiotics in the GIT, which of these differences relate to the growth of the bacteria, and whether acid stress contributes to these alterations. Our results indicate that fermented sugar regulates peptidoglycan homeostasis, biofilm formation, and antimicrobial production and, therefore, could alter the competitiveness of LGG in the competitive GIT.

## Results

### The effect of fermentable sugars on proteome architecture

**Fig. 1:**
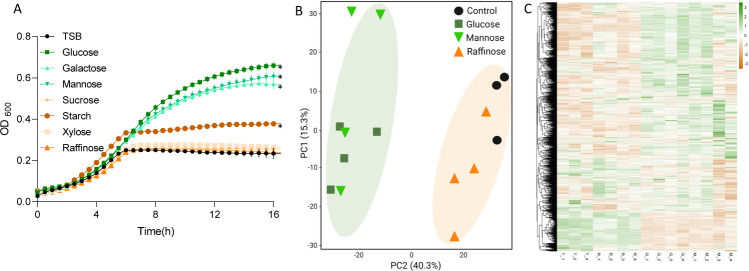
The effect of glucose, mannose, and raffinose on the growth and proteome of LGG. **A** Growth curves of LGG in 96 well plates at 37 °C with shaking in TSB medium or TSB medium supplemented with 1% W/V of different sugars. Statistical analysis was performed using one-way ANOVA with Tukey’s post-hoc test vs. TSB. *p* < 0.05 was considered statistically significant. **B** Principal component analysis (PCA) plot of proteomics analysis of LGG grown in liquid TSB medium [T1-T4] or TSB medium supplemented with glucose (1% W/V) [G1-G4], mannose (1% W/V) [M1-M4] or raffinose (1% W/V) [R1-R4]. **C** Heat map showing the mass-spec intensities of all the proteins identified in proteomics analysis across conditions.

We previously found that fermentable sugars specifically affect the probiotic bacterium LGG, functioning as distinct regulators of growth, aggregation, and adhesion^12^. The application of glucose, galactose, and mannose, which can be fermented by LGG^13^, induced growth compared to the tryptic soy broth (TSB) control medium. Here, we extended the repertoire of consumed carbohydrates in LGG. The application of starch increased the growth of LGG to a lesser extent compared with glucose, galactose, and mannose. Starch is composed of glucose units joined by the glycosidic bond, and this result indicates that LGG can only partially degrade starch into glucose. However, non-fermentable sugars such as sucrose, xylose, and raffinose did not induce the growth of LGG in shaking cultures (Fig. 1A). The growth with glucose and mannose was enhanced with buffer (Fig. S1), while the growth with raffinose was not (Supplementary Fig. 1). This result is indicative of accumulation of acidic products exerting some level of toxicity to the bacterium.

The effects of fermentation on bacterial growth and pH are well established. However, the metabolic status of bacteria in the GIT can also affect gene expression, and therefore the protein architecture. An unbiased proteomics analysis was performed to test the global response of LGG to fermentation and to identify pathways that are changed following fermentation, either directly by metabolite-transcription interactions, or by the altered carrying capacity obtained with glucose and mannose (Fig. 1A). To the best of our knowledge, this is the first proteomic evaluation of pathways acting downstream to fermentation on LGG. Principal component analysis (PCA) of the proteomic data demonstrated that the protein profiles of LGG in fermentation (application of glucose and mannose), are clustered closely together but both partition from the proteome of non-fermenting cultures (application of raffinose or TSB control) with the second component (PCA2) explaining 40% of the data variance (Fig. 1B). This result is also reflected in the heat map, where two major clusters separating between fermentation and non-fermentation conditions can be seen (Fig. 1C, Supplementary Table 1). We determined that these changes are triggered by fermentation and not specifically by glucose as a carbon source, as both mannose and glucose are converted into fructose-6-phosphate and proceed to the glycolysis pathway^11^.

### Global metabolic changes associated with altered growth and fermentation

**Fig. 2:**
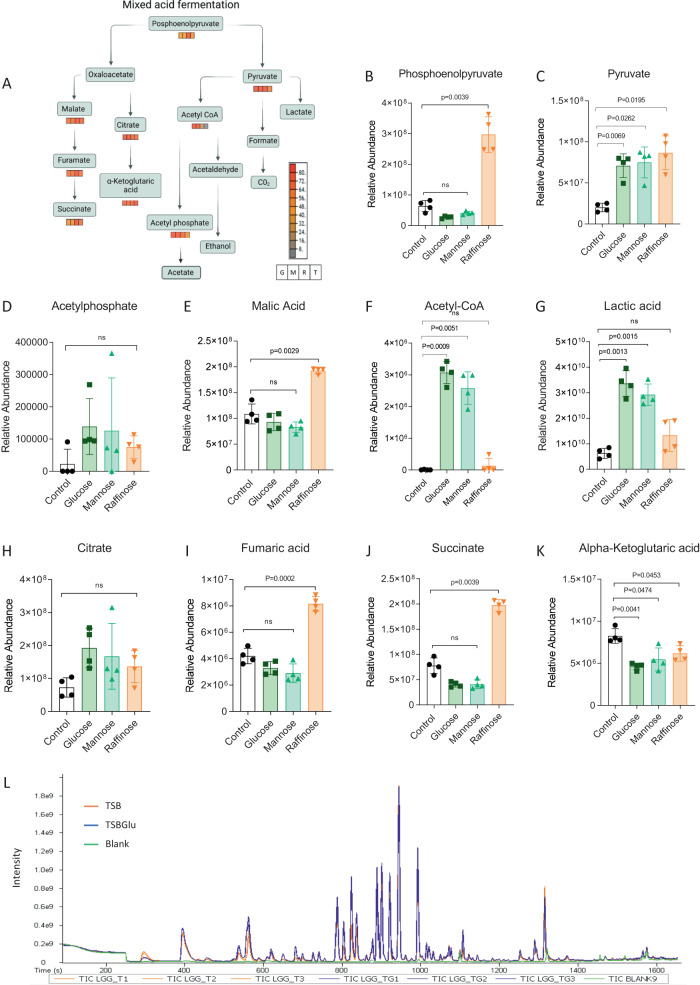
LGG metabolite profile is affected by fermentation. **A** MetaCyc metabolic pathway of mixed acid fermentation pathway based on metabolomics analysis from LGG grown in TBS medium or TSB medium with glucose (1% W/V), mannose (1% W/V), or raffinose (1% W/V). Relative abundance of **B** Posphoenolpyruvate, **C** Pyruvate, **D** Acetylphosphate, **E** Malic acid, **F** Acetyl-CoA, **G** Lactic acidt, **H** Citerate, **I** Fumaric acid, **J** Succinate, and **K** Alpha-keto glutaric acid. The images represent 4 independent repetitions. Statistical analysis was performed using Brown-Forsythe and Welch’s ANOVA with Dunnett’s T3 multiple comparisons test. *p* < 0.05 was considered statistically significant. **L** Chromatographic profile obtained by GC-MS of volatile compounds from LGG grown in TBS medium or TSB medium supplemented glucose (1% W/V).

To increase our resolution of the response of LGG to the metabolic variations, we performed untargeted metabolomics on LGG grown with and without glucose and mannose. As judged by PCA, LGG cultured with fermentable sugars clustered separately from the control media and raffinose supplemented media, with the first component, explaining 44.5% of the data variance (Supplementary Fig. 2, Supplementary Table 2). These results correlate with our proteomics results with the control and raffinose clustering together, but separately from glucose and mannose (Figs. 1B and 2). The MetaCyc metabolic pathway toolkit^14^ indicated that metabolites from the mixed acid fermentation pathway changed significantly in response to the carbohydrate source (Fig. 2A). Pyruvate abundance in LGG cultured with glucose and mannose was significantly higher than with un-supplemented media. However, pyruvate also increased with raffinose, suggesting that under our experimental conditions, pyruvate is not directly affected by fermentation (Fig. 2C). Both lactic acid and acetyl-CoA were significantly induced with glucose and mannose (compared with TSB control), but not with raffinose (Fig. 2F, G). This is consistent with our growth analysis indicating that buffering enhances the growth only when the bacteria are provided with glucose and mannose. Metabolites associated with glycolysis (Supplementary Fig. 3) were also altered, with no consistent trends between the different metabolites, e.g., D-Glyceraldehyde 3-phosphate levels were increased with glucose and mannose, but 3-phosphoglycerae levels were comparable in all conditions except raffinose. In general, variation was observed in the accumulation of fermentation associated core metabolites with the different carbohyrates (Fig. 2A–K).

Changes in intracellular metabolome also affect the air borne metabolites that mediate interspecies interactions from afar, termed volatiles. To test whether extracellular volatiles are affected by carbohydrate consumption and how, we systematically analyzed the LGG volatilome, considering the effects of fermentation on this process. We grew LGG in liquid TSB or TSB applied with glucose and sampled the volatile compounds (VCs) in the headspace above the cultures using a GC-TOF-MS. Consistent with the metabolic rewiring of the metabolome and proteome, fermentation altered the VC profile overall and induced the production of organic volatiles (Fig. 2L, Supplementary Table 3). In the TSB medium, background from the media was evident for multiple volatiles, complicating the analysis. However, 3-Methylbutanol was definitely increased by fermentation (Supplementary Fig. 4).

### Specific changes in cell wall biosynthesis are induced by fermentable sugars

**Fig. 3:**
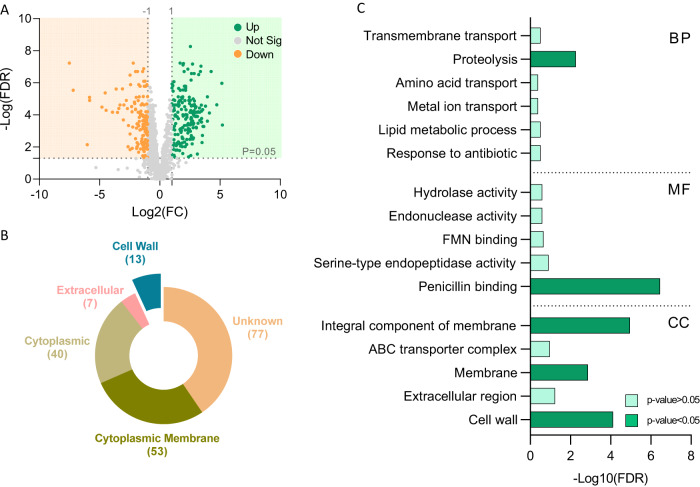
The abundance of cell-wall biosynthesis and structural proteins is specifically altered by fermentable carbon sources. **A** Volcano plot representing the log2 fold-change (fermentation/non-fermentation) against −log10 statistical *p*-value with FDR correction for proteins differentially expressed between glucose and mannose (fermentation) vs. control and raffinose (non-fermentation). Gray dots represent unchanged proteins, green and orange dots represent the upregulated and downregulated proteins, respectively; the horizontal dashed line indicates FDR = 0.05 and vertical dashed line indicates log2FC >|1|. **B** Pie chart represent PSORT analysis and **C** Bar chart shows Over Representation Analysis (ORA-WebGestalt) of significantly upregulated proteins during fermentation.

We demonstrated that the intracellular and extracellular metabolome is rewired during fermentation and an enhanced intracellular accumulation of acidic products (Fig. 2F, G). Thereby to adapt to fermentable sugars, the bacteria are expected to increase their overall tolerance to cell envelope damage. Furthermore, in our previous work, we could detect membrane damage triggered by glucose^12^. Our proteome analysis also indicated global changes in peptidoglycan. About 195 out of 1800 proteins were significantly more abundant when the fermentable sugars glucose and mannose were applied, and about 140 proteins were significantly less abundant (Fig. 3A and Supplementary Table 1). Therefore, we employed PSORT analysis and the web-based Gene Set Enrichment Analysis Toolkit^15^ to explore potential microbial adaptations to the envelope stress exerted by fermentation products. Our analysis revealed that the cell wall biosynthesis and turnover components are induced by fermentation (Fig. 3B, C). The Gram-positive bacterial cell wall is composed of layers of peptidoglycan, a polymer made from polysaccharide chains cross-linked by peptides containing D-amino acids and layers of teichoic acid^16^. The categories of cell wall proteins that were altered include penicillin-binding proteins, which are generally involved in peptidoglycan biosynthesis^17^.

**Fig. 4:**
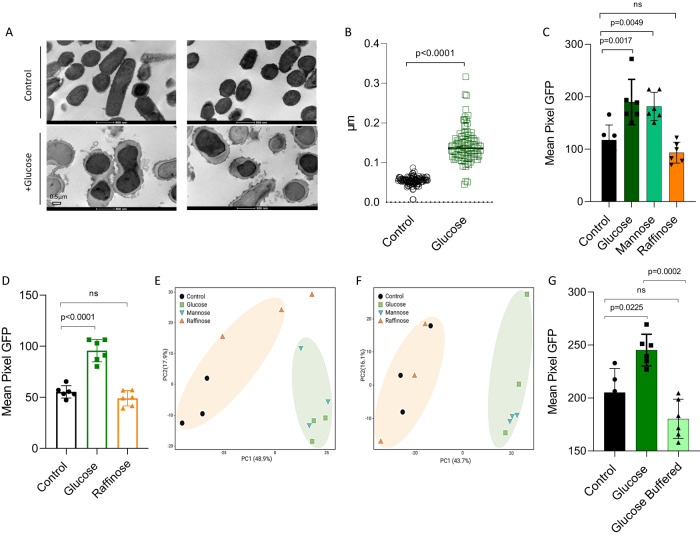
The Structure and composition of the cell wall is altered during fermentation. **A** Transmission electron microscopy (TEM) images of LGG colony grown in solid TSB medium (control) or TSB medium supplemented with glucose (1% W/V). Scale bar = 0.5 μm. **B** Quantification of 100 cells’ cell wall thickness from TEM images using Fiji-ImageJ. Statistical analysis was performed using Unpaired *t* test with Welch’s correction. *p* < 0.05 was considered statistically significant. **C**, **G** Imaging Flow cytometry analysis of the mean pixel intensity of LGG cells that were grown on solid TSB (control), TSB supplemented with glucose (1% W/V), mannose (1% W/V), raffinose (1% W/V) or TSB medium supplemented with glucose (1% W/V) + buffer. Cells were labeled using WGA-FITC. Biofilms were grown at 37 °C in a CO_2_ enriched environment. Data were collected after 72 h, and 100,000 cells were counted. Graphs represent mean ± SD from 2 independent experiments (*n* = 6). All statistical analysis was performed using Brown-Forsythe and Welch’s ANOVA with Dunnett’s T3 multiple comparisons test. *p* < 0.05 was considered statistically significant. **D** Imaging flow cytometry analysis of the mean pixel intensity of LGG cellss that were grown on solid TSB (control) and TSB supplemented with glucose (1% W/V) or raffinose (1% W/V). Cells were labeled using BODIPY™ FL vancomycin^21^. Biofilms were grown at 37 °C in a CO_2_ enriched environment. Data were collected after 72 h, and 50,000 cells were counted. Graphs represent mean ± SD from 2 independent experiments (*n* = 6). Statistical analysis was performed using Brown-Forsythe and Welch’s ANOVA with Dunnett’s T3 multiple comparisons test. *p* < 0.05 was considered statistically significant. **E** PCA analysis comparing between treatments of biofilm-derived peptidoglycan and **F** planktonic-derived peptidoglycan. The images represent 3 independent repetitions.

To further evaluate the response of the cell wall to fermentation, we manually screened for cell-wall proteins that may affect the competitiveness of LGG in the GIT and are affected by fermentation. We found that a class A Sortase (Supplementary Fig. 5) was induced by fermentation. This membrane-associated cysteine transferase belongs to a family of proteins that facilitates the bacterial evasion of the host’s immune system^18^ and is involved in immunomodulation, adhesion to epithelial cells, and antibacterial activity against gut pathogens^19^. In our previous publication^12^, we found that LGG cells grown in colonies in the absence of fermentable sugar are rod-shaped and clearly separated. In contrast, colony cells grown with fermentable sugar lost the elongated rod shape. To understand whether the cell wall morphology also changed in cells grown with a fermentable sugar, we looked at the cells under a transmission electron microscope (TEM), which allowed us to resolve cellular structures by performing a cross-section of the bacterial cells. The cells grown without glucose had a rod shape with smooth edges. In contrast, cells grown on glucose had a rounder, atypical shape, consistent with their appearance in the light microscope. The edges were not smooth but rather laced (Fig. 4A). We suggest that the changes that we observed in the proteome may explain the underlying mechanism for the observed changes in the cells. A quantification of cell wall thickness cell from TEM images (Fig. 4B) confirmed this adaptation, consistent with enhanced tolerance to antimicrobials^20^. In addition, image stream flow cytometry analysis with two fluorophores, wheat germ agglutinin (WGA), that binds N‐acetylglucosamine, and subtoxic concentration of vancomycin-BODIPY FL^21^ labeling the newly synthesized muramopeptide also indicated significant alterations in the cellular envelope (Fig. 4C, D). To confirm that this effect is growth-independent, we repeated this analysis in a shaking culture, at early time points where growth was not affected (Supplementary Fig. 6). Our results confirmed that the presence of glucose alters the synthesis of the muramopeptide prior to detectable changes in cell growth.

To further confirm that the altered levels of cell wall biosynthesis proteins during fermentation reflect physiological changes in peptidoglycan composition, we analyzed muropeptide composition using LC-MS during both planktonic growth and biofilm formation. Our analysis demonstrated a large distinction in cell composition during the application of fermentable sugars, independently of the lifestyle of the bacteria (Fig. 4E, F).

To determine whether cell wall reorganization is induced by acidic fermentation products, we added buffer to the growth media. The buffer restored the basal level of PG staining (Fig. 4G), indicating that the alterations in the peptidoglycan were caused by the pH drop.

**Fig. 5:**
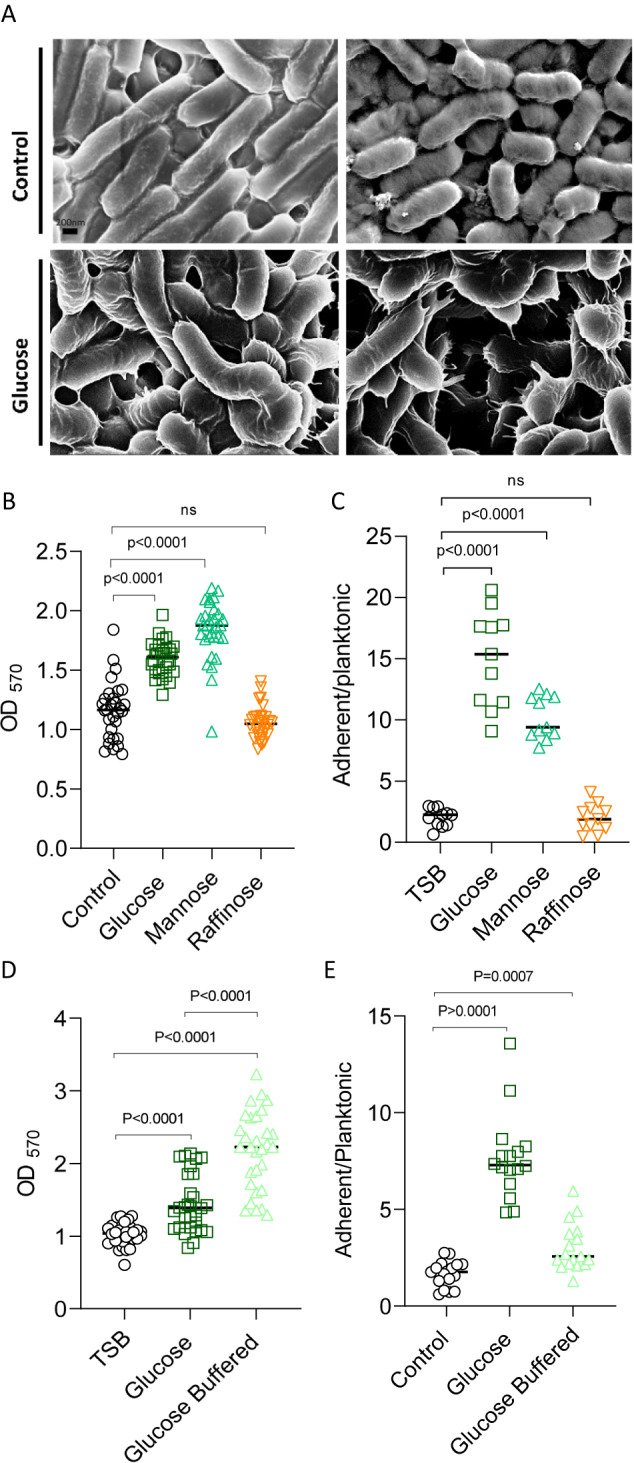
Biofilm formation is enhanced in response to fermentable carbohydrates. **A** Scanning electron microscopy (SEM) images of LGG grown in solid TSB (control) and TSB supplemented with glucose (1% W/V). The colonies were incubated at 37 °C in an environment enriched with CO_2_ for 3 days. **B**, **D** LGG cells were diluted 1:100 into fresh TSB medium or TSB supplemented with glucose (1% W/V), mannose (1% W/V), raffinose (1% W/V), or TSB medium supplemented with glucose (1% W/V) + buffer. 200 μL of cultures were split into a 96-well polystyrene plate and further incubated at 37 °C. Crystal violet assay assessed the biofilm formation of LGG after 72 h. Graph represents the mean ± SD from three biological repeats (*n* = 30). **C**, **E** LGG cells were diluted 1:100 into a fresh TSB medium or TSB supplemented with glucose (1% W/V), mannose (1% W/V), raffinose (1% W/V), or TSB medium supplemented with glucose (1% W/V) + buffer. 1.5 mL of cultures were split into a 12-polystyrene plate and further incubated at 37 °C for 24 h. The upper growth media was removed and OD_600_ measured. The remaining biofilm biomass was diluted in fresh TSB and OD_600_ measured. *Y* axis represents the ratio of OD_600_ (planktonic/biofilm biomass). Graph represents the mean ± SD from three biological repeats (*n* = 12). All statistical analysis was performed using Brown-Forsythe and Welch’s ANOVA with Dunnett’s T3 multiple comparisons test. *p* < 0.05 was considered statistically significant.

In non-LAB bacteria, it was demonstrated that cell wall homeostasis is tightly linked to biofilm formation^21,22^ and is frequently co-regulated with the extracellular matrix genes^23–25^. Therefore, we examined biofilm formation across the tested carbohydrates. Indeed, glucose and mannose, but not raffinose, significantly induced biofilm formation as judged by the biofilm biomass that accumulated on the cell surface (Fig. 5B) and the ratio of surface-associated and free-living cells (Fig. 5C). Under these conditions, the colony was flat and featureless. Bacteria positioned at the edges of the colony swarmed [collectively migrated over the agar surface], and therefore, the edges of the colony were not symmetrical. The application of raffinose had little or no effect on colony morphology. However, the application of glucose and mannose induced the formation of symmetric and thick colonies (Supplementary Fig. 7). The enhanced production of the extracellular matrix was evident in LGG colony cells grown with glucose, demonstrating an increased production of the extracellular matrix compared with TSB alone (Fig. 5A). Glucose and mannose also induced proteins related to adhesion and biofilm formation^26,27^ (Supplementary Fig. 8). Interestingly, the fermentation-dependent changes in the muramopeptidome were observed during biofilm formation (Fig. 4E, F), indicating that the cell wall peptidome is not altered by fermentation-dependent biofilm formation but is rather a direct readout of fermentation. Consistent with a common regulation of biofilm formation and cell wall reorganization by the pH drop, buffer also restored basal levels of biofilm formation to the culture (Fig. 5D, E). Notably planktonic growth was enhanced with buffering (Fig. S1), while biofilm formation was not significantly altered (Fig. 5D, E), indicating that the acid-imposed stress of fermentation can be beneficial for cell-cell adhesion and surface adhesion. In addition, the significant reduction of cell wall thickening (as judged by image stream flow cytometry) with buffering, and the lack of significant response of bacterial biofilms indicate that these processes may occur independently. Similar to the cell wall remodeling, the adhesion of LGG cells to mucin^12^, a property indicative of their capacity to express biofilm adhesins (and to adhere to a mammalian host), was significantly altered by glucose prior to detectable changes in cell growth (Supplementary Fig. 9).

### Fermentation drives the secretion of potent organic acids and protease-sensitive effectors

**Fig. 6:**
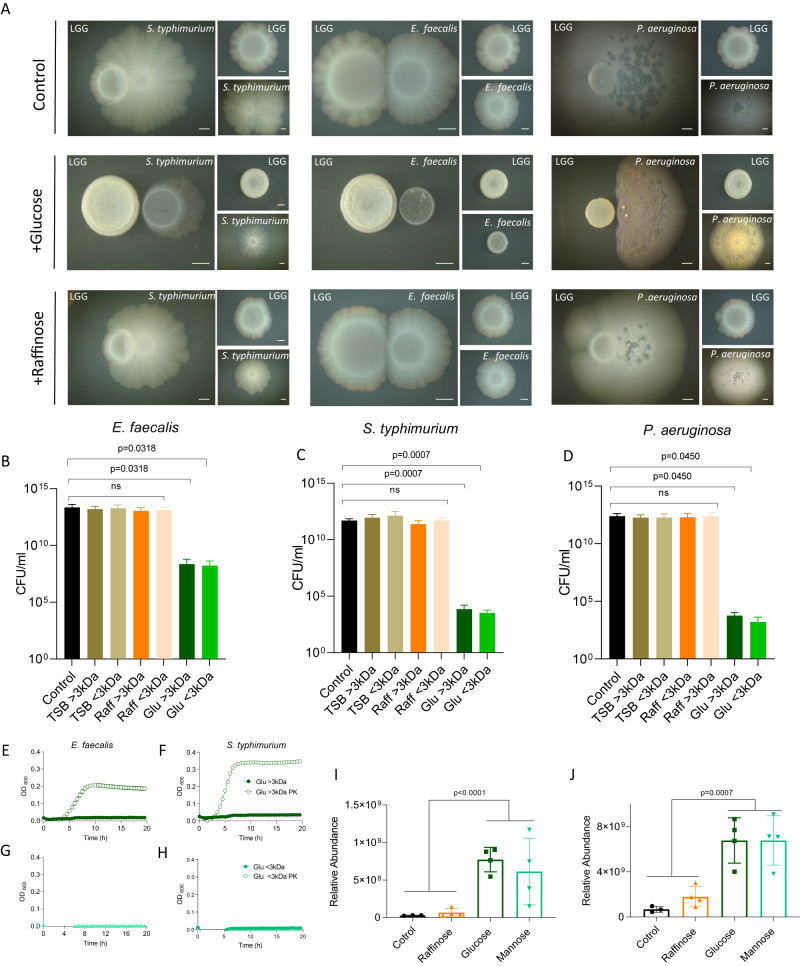
Fermentation has a cardinal role in antimicrobial production. **A** Colonies of LGG, *S. typhimurium, E. faecalis, and P. aeruginosa*, grown on solid TSB medium or TSB supplemented with glucose (1% W/V) or raffinose (1% W/V). The colonies were incubated at 37 °C, in a CO_2_ enriched environment for 7 days. Colonies of LGG were inoculated next to *S. typhimurium*, *E. faecalis,* or *P. aeruginosa* colonies at a distance of 0.2 cm. **B**–**D** Colony-forming unit counts of *E. faecalis* or *S. typhimurium* or *P. aeruginosa* grown in 30% CM > 3 kDa or <3 kDa from LGG grown in TSB medium or TSB supplemented with glucose (1% W/V) or raffinose (1% W/V). Statistical analysis was performed using Brown-Forsythe and Welch’s ANOVA with Dunnett’s T3 multiple comparisons test. *p* < 0.05 was considered statistically significant. Growth curves of **E** *E. faecalis* and **F** *S. typhimurium* in 96 well plates in 37 °C with shaking. Cells were supplemented with 30% CM > 3 kDa from LGG grown in TSB medium supplemented with glucose (1% W/V) or 30% CM > 3 kDa from LGG grown in TSB medium supplemented with glucose (1% W/V) after proteinase K treatment (PK). Growth curves of **G** *E. faecalis*, **H** *S. typhimurium* in 96 well plates at 37 °C with shaking. Cells were supplemented with 30% CM < 3 kDa from LGG grown in TSB medium supplemented with glucose (1% W/V) or 30% CM < 3 kDa from LGG grown in TSB medium supplemented with glucose (1% W/V) after proteinase K treatment (PK). **I** Protein relative abundance between the sample of Msp1/p75 and **J** Msp2/p40. Statistical analysis was performed using Student’s *t* tests followed by FDR correction. *p* < 0.05 was considered statistically significant.

To examine whether LGG secretes proteinaceous antibacterial substances together with organic acids during fermentation, we tested whether fermentation could alter the outcomes of microbial competition of LGG against enteric pathogens. LGG colonies grown with fermentable glucose in the medium were more competitive in the presence of gut pathogens. In the absence of glucose or application of non-fermentable raffinose, *S. typhimurium* colonies attached to and engulfed LGG colonies. Under these conditions, *E. faecalis* colonies merged with and invaded colonies formed by LGG (Fig. 6A). In contrast, in the presence of glucose, *S. typhimurium and E. faecalis* colonies were strongly antagonized by LGG during competition (Fig. 6A). In addition, we tested the competitiveness of LGG during fermentation against additional bacteria that do not share the same niche, i.e., *P. aeruginosa*. Consistent with the increased broad-spectrum effects versus enteric pathogens, *P. aeruginosa* was clearly antagonized by LGG in a fermentation-dependent manner (Fig. 6A).

The inhibitory product was secreted as predicted by the proteome as the conditioned medium of LGG with glucose (but not glucose) inhibited the growth of all three pathogens (Supplementary Fig. 10). Conditioned medium of LGG stationary cultures in the absence of fermentable sugar had little or no effect on the growth of these competitors. To confirm a fermentation-dependent proteinous component in the toxicity of LGG, we separated the condition medium by size of 3 KDa to separate small molecules (primarily organic acids and proteins). Fractions bigger and smaller than 3 kDa obtained from conditioned medium with glucose significantly inhibited the growth all three pathogens while fractions from conditioned media collected with no added sugar or raffinose had a significantly reduced activity (Fig. 6B–D). The proteome analysis supported the notion of antibacterial protein effectors being induced during fermentation. The role of fermentation-induced proteins was further supported by the protease sensitivity of the antimicrobial properties of the >3 kDa fraction (Fig. 6E, F). In contrast, the antimicrobial activity of the smaller fraction, <3 kDa, was protease resistant (Fig. 6G, H)

The proteome indicated a potential role for Msp1/p75 (Fig. 6I) and Msp2/p40 (Fig. 6J) that were induced by fermentation. Msp1/p75 was also detected independently in the >3KDa fraction by MS analysis (Supplementary Fig. 11). These proteins are secreted by LGG^28^ and are probiotic effectors^29^, with peptidoglycan hydrolase activity^30^. Msp1/P75 was characterized as a d-glutamyl-l-lysyl endopeptidase with a role in cell wall metabolism^31^, has an inhibitory effect on *Candida albicans* by chitinase activity^32^, and surface display of this protein in *Bacillus subtilis* enhanced its antibacterial activity against *Listeria monocytogenes*^33^. However, we cannot exclude the chance that additional proteins play a role in the fermentation-dependent antimicrobial secretome.

### Subtoxic levels of acetic acid and acetyl-CoA contribute to proteome modification

To test whether acetic acid (and thereby also acidity per se) can mimic the effects of fermentable glucose and mannose, we repeated our proteome analysis with subtoxic levels of acetate. While our data support the notion that the graduated accumulation of organic acids are slightly toxic to LGG (Supplementary Fig. 1), the net effect of glucose provided early during growth is inducing, and thereby cannot be compared easily to the final toxic concentration of the generated core metabolites (Supplementary Figs. 12–14).

**Fig. 7:**
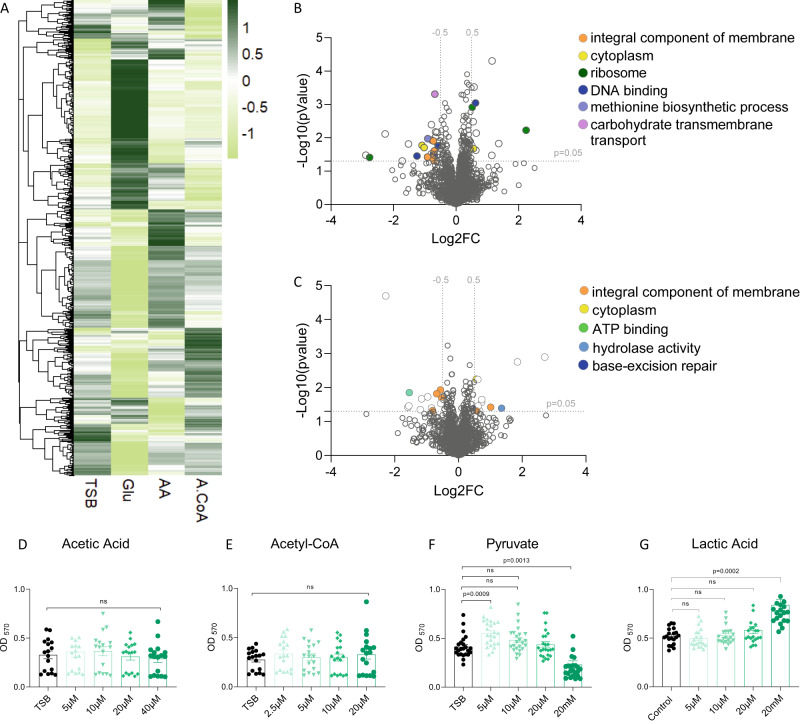
The effect of core metabolites generated during fermentation of the preome architecture, and biofilm formation. **A** Heat map showing the mass-spec intensities of all the proteins identified in proteomics analysis of LGG grown in liquid TSB medium, TSB medium supplemented with glucose (1% W/V), TSB medium supplemented with 20 µM acetic acid (AA) or TSB medium supplemented with 10 µM acetyl-CoA (A-CoA). Volcano plot representing the log2 fold-change (AA/TSB) against -log10 statistical *p*-value for proteins differentially expressed between (**B**) acetic acid vs. control (TSB) and **C** acetyl-coA vs. control (TSB). Colored dots represent the GO categories of differentially expressed proteins. The horizontal dashed line indicates *p* = 0.05 and vertical dashed line indicates a log2FC of |0.5|. **D** LGG cells were diluted 1:100 into a fresh TSB medium or TSB supplemented with different concentrations of acetic acid, **E** acetyl-CoA, **F** pyruvate, and **G** lactic acid. 200 μL of cultures were split into a 96-well polystyrene plate and further incubated at 37 °C. Crystal violet assay was used to assess the biofilm formation of LGG after 72 h. Graph represents the mean ± SD from three biological repeats. Statistical analysis was performed using Brown-Forsythe and Welch’s ANOVA with Dunnett’s T3 multiple comparisons test. *p* < 0.05 was considered statistically significant.

Indeed, subtoxic acetic acid levels altered the proteome of LGG (Fig. 7A, B, and (Supplementary Table 4) and significantly altered the levels of 45 proteins. Acetic acid induced twelve proteins and repressed 32 proteins, including several transcription factors from the *marR* family (WP_005686521.1) and a transcriptional repressor (WP_005687049.1) from the TetR/AcrR family. TetRs act as chemical sensors to both monitor the cellular environmental dynamics and regulate genes responsible for antibiotic production, osmotic stress, efflux pumps, multidrug resistance, and metabolic modulation^34^. This result indicates that subtoxic acetate levels may be sufficient to trigger cellular stress. Acetate also induces a phage protein, a tail assembly chaperon (WP_014569494.1), which may indicate intracellular stress as phages are often induced by damaging essential cellular components such as DNA integrity^35^. In addition, acetate significantly altered the levels of multiple transporters, including citrate sympoter (WP_005686012.1) and PTS sugar transporters (WP_014569015.1), and induced GNAT family N-acetyltransferase (WP_005714043.1), from the FabZ superfamily (WP_005684766.1), involved in maltose catabolism^36^.

In addition to acetate, the levels of acetyl-CoA were elevated in the presence of glucose and mannose but not raffinose (Fig. 2F), and thereby, altered similarly to the overall proteome. This nonacidic core metabolite (generated from pyruvate in mixed acid fermentation) induced a specific response in 25 proteins, distinctive of both glucose and acetic acid (Fig. 7A, C, Supplementary Table 4). Acetyl-CoA also significantly induced the levels of 12 proteins, and thereby induced a more modest effect compared to acetate. Acetyl-CoA induced the levels of aminobenzoyl-glutamate utilization protein B (WP_014569109.1) and a sodium channel (WP_005686017.1), as well as two families of proteins, conserved in *Firmicutes* (Duf3042, DUF956 and DUF956) with an unknown function. In all cases, these proteins were not induced by glucose. Interestingly, acetyl-CoA repressed a histidine kinase (WP_014569412.1), previously reported as responsible for the modulation of quorum sensing by acetic acid^37^. Acetyl-CoA was also associated with an altered metal homeostasis as both a heavy metal translocating P-type ATPase (WP_014568961.1) and a putative metal homeostasis regulator (WP_015765004.1) were significantly repressed.

Overall, our results linked organic acids to altered cell wall homeostasis, altered antimicrobial activity, and acetyl-CoA with specific pathways whose role in biofilm formation and cell wall homeostasis remains to be determined. A direct assessment of biofilm formation with pure acetic acid, or acetyl-CoA revealed little or no effect of these metabolites (Fig. 7D, E). However, we found that pyruvate is capable of both inducing (at 5 µM) and repressing (at 20 mM) biofilm formation (Fig. 7F). Pyruvate levels were enhanced in the presence of both glucose and raffinose, while cellular adhesins were only induced in the presence of glucose and mannose. Lactate was also capable of significantly inducing biofilm formation (Fig. 7G) but only in toxic concentrations. Therefore, the specific metabolic pathway linking glucose with enhanced biofilm formation involved pyruvate and lactate, but must be further explored.

Overall, these results indicate that the metabolic response to fermentable sugars may be intertwined and cannot be replicated with continuous growth in the presence of a single product of fermentation.

## Discussion

The structure and function of microbiome communities are greatly affected by the availability of trace nutrients, carbon, and nitrogen sources. The gastrointestinal tract primarily provides these nutrients from the host’s diet^38^. The subsequent analysis of alterations in the gut microbiota, comparing the effects of multiple environmental factors, including nutrients, suggests that the altered microbial communities influence the liver and adipose tissues, altering their metabolic state^39^. The effects of the human microbiome on the host’s metabolism are extensive and include but are not limited to food intake, digestion, absorption, and the production of a broad repertoire of metabolites. These metabolites include bile acids, amino acids and their derivatives, and short-chain fatty acids, which were shown to play a fundamental part in GIT homeostasis^40,41^. Microbiota-derived metabolites also regulate the intestinal absorption and lipid metabolism within the intestine, and this effect is attributed mainly to organic acids^42^.

Probiotic bacteria are a subset of GIT microbiota proposed to benefit human health. Here we use the probiotic bacterium LGG to link between simple fermentable carbohydrates, microbial physiology, and microbial metabolites. The health benefits of LGG include preventing and treating gastrointestinal infections and diarrhea and stimulating immune responses that promote vaccination or even prevent specific allergic symptoms. However, only some intervention studies have demonstrated the clinical benefit of probiotics, and even for the same conditions, the results are only sometimes consistent^29^. By linking fermentable sugars with LGG performance in vitro, we can generate focused predictions regarding specific dietary-associated probiotic behavior. First, probiotics can exclude or inhibit pathogens through direct action or influence on the commensal microbiota^43–45^.

We show here that the availability of fermentable sugar is a cardinal determinant of this behavior and can be attributed to enhanced accumulation of cytotoxic metabolites and volatiles (organic acids and butanol, Fig. 2 and Supplementary Fig. 4), and antimicrobial extracellular proteins (Fig. 6E, F). While a broad spectrum of pathogenic bacteria is eliminated by LGG in a fermentation-dependent manner (Fig. 6A), the bacterium itself alters its cell wall (Fig. 4) and biofilm formation (Fig. 5B, C), as a potential coping mechanism against the same conditions. Furthermore, our results indicated that cell wall remodeling precedes biofilm formation, and one appealing hypothesis is that the accumulation of toxic fermentation products^12^ triggers the cell wall remodeling. Also supporting this notion, is that one of the antimicrobial proteins induced during fermentation (Fig. 6I) is a d-glutamyl-l-lysyl endopeptidase, targeting peptidoglycan. The cell wall can be considered a readout of an environmental pH drop as it was restored to basal levels with buffering (Fig. 4G). However, the levels of cell wall remodeling enzymes could not be restored with supplementation of subtoxic concentrations of acetic acid to the growth media, indicating that these processes may not be a simple readout of the pH of the media. One difficulty in assessing the input of each metabolite is their complicated effects on growth. While the growth rate is comparable with raffinose, mannose, and glucose^12^, a differential carrying capacity is observed, and (near-) zero growth rates affect the proteome and metabolome^46^. However, carefully examining the curves suggests that the growth is also feeble, with glucose and mannose from the 12^th^ hour of growth. Therefore, all cultures were analyzed for their proteome and metabolome of prolonged stationary phase. This status best reflects the growth in the GIT, where many beneficial bacteria are in a biofilm state and thereby are slowly replicating or at the stationary stage^47^. An additional consideration is the toxicity of glucose catabolites when these acidic catabolites are provided, even at sub-physiological concentrations before the gradual adaptation. A gradual pH drop could significantly differ from acute acid stress, allowing the fundamental adaptation of multiple processes reflected by the proteome remodeling. In addition, biofilm formation could not be restored to the basal levels by buffering (Fig. 5D, E). It could be induced (by a subtoxic concentration) or repressed (by toxic concentrations) by pyruvate (Fig. 7F) and also induced by high concentrations of lactic acid (Fig. 7G).

Biofilm formation is tightly linked with cell wall remodeling and is expected to be co-regulated with antimicrobial activity^48,49^. Furthermore, acetate (accumulated intracellularly and emitted as a volatile) activates the biosynthesis of the bacteriocin rhamnosin B in LGG when applied as acetic acid salt^37^. Consistently, our data provide strong evidence that cell wall remodeling precedes an induced biofilm formation, which does not depend on organic acids (Fig. 5), and these behaviors are correlated with enhanced antimicrobial production (Fig. 6). Unlike acetic acid and acetyl-CoA, pyruvate was found to both induce and repress biofilm formation in a concentration dependent manner. Biofilm induction was not a mere reflection of growth as the ratio between planktonic cell numbers and adherent cells was also altered during biofilm formation (Fig. 5C, E).

As pyruvate levels fluctuated in the presence of both fermentable glucose and mannose, but also raffinose, the exact role played solely by pyruvate in biofilm formation remains to be determined.

A second mechanism by which LGG exerts host-beneficial effects is modulating host immune responses, exerting strain-specific local and systemic effects^50^. Microbe-associated molecular patterns (MAMPs) through specific pattern recognition receptors, including toll-like receptors (TLRs). These receptors mediate many of the interactions between probiotic bacteria, the immune cells, and the intestinal epithelium^50–53^. The behaviors of microbiome members greatly depend on the capacity of a bacterium to adhere to host tissues manifested in enhanced biofilm formation, which occurs downstream to fermentation (Fig. 5A–C) and the fermentation-dependent expression of proteins associated with increased adhesion (Supplementary Fig. 8). Interestingly our results indicate that different core metabolites generated downstream to glucose catabolism can be linked to antimicrobial activity, biofilm formation and cell wall remodeling.

In vitro data and experiments were validated by numerous animal models that confirmed diet-bacterial response-host interactions for probiotic strains, including LGG. However, most published data are from in vivo studies in humans with poor detection of the bacterial mechanisms of action, with a “black-box” regarding the means of interaction with the host and the host’s diet. Here we provide compelling evidence linking specific carbohydrates and their catabolites with the performance of probiotic strains and identify specific cellular processes associated with the different downstream catabolites of glucose. Our results may yield improved interventions based on probiotic bacteria or their products.

## Methods

### Strains, media and imaging

*Lacticaseibacillus rhamnosus* GG (LGG) ATCC 53103 was the probiotic strain used in this study. *Enterococcus faecalis* ATCC 29212142, *Salmonella enterica* Serovar Typhimurium [kindly provided by Dr. Roi Avraham’s Lab] and *Pseudomonas aeruginosa* PA14 were used for competition assay. A single colony of *L. rhamnosus* GG was isolated on a solid deMan, Rogosa, Sharpe (MRS) plate (1.5% agar), inoculated into 5 mL MRS broth (Difco, Le Pont de Claix, France), and grown at 37 °C, without shaking overnight. A single colony of *E. faecalis, S. typhimurium,* and *P. aeruginosa, respectively,* isolated on a solid LB agar plate was inoculated into 3 mL Luria-Bertani (LB) (Difco) and grown at 37 °C, with shaking overnight. For biofilm colonies, these cultures were inoculated into a solid medium (1.5% agar) containing 50% tryptic soy broth (TSB), TSB supplemented with (1% w/v) D-(+)-glucose, (1% w/v) D-(+)-raffinose, (1% w/v) D- (+)-mannose or (1% w/v) D-(+)-glucose buffered with MOPS (3-(N-morpholino)propane-sulfonic acid) and potassium phosphate buffer. The bacteria were incubated in a BD GasPak EZ-Incubation Container with BD GasPak EZ CO_2_ Container System Sachets (260679) (Becton, Sparks, MD, USA), for 72 h or 7 days, at 37 °C. The colony images were taken using a Stereo Discovery V20” microscope (Tochigi, Japan) with objectives Plan Apo S ×1.0 FWD 60 mm (Zeiss, Goettingen, Germany) attached to a high-resolution microscopy Axiocam camera. Data were generated and processed using Axiovision suite software (Zeiss). For planktonic growth, the bacterial cultures were inoculated into a liquid medium of 50% TSB with different sugars as described above, incubated for 24 h, with no shaking, at 37 °C.

### Growth measurement and analysis

LGG cultured cells grown overnight were diluted 1:100 in 200 μL liquid medium containing 50% TSB (BD) or TSB supplemented with (1% w/v) different sugars in a 96-well microplate (Thermo Scientific, Roskilde, Denmark). *S. typhimurium, E. faecalis, and P. aeruginosa* cultured cells grown overnight were diluted 1:100 in 200 μL liquid medium containing TSB supplemented with (1% w/v) and with 30% conditioned medium derived from LGG grown with and without sugars, as mentioned in the first section. For LGG growth with metabolites, acetic acid, acetyl-coA and pyruvate were added to TSB liquid medium in in different concentrations. Cells were grown with agitation at 37 °C for 16–20 h in a microplate reader (Tecan, Männedorf, Switzerland), and the optical density at 600 nm (OD_600_) was measured every 30 min.

### Fluorescence microscopy

A bacterial biofilm colony of LGG grown as described above was suspended in 200 μL 1× phosphate-buffered saline (PBS), and dispersed by pipetting. Samples were centrifuged briefly, pelleted, and re-suspended in 5 μL of 1× PBS supplemented with the membrane stain FM1-43 (Molecular Probes, Eugene, OR, USA) at 1 μg/mL. The cells were then placed on a microscope slide and covered with a poly-L-Lysine (Sigma) treated coverslip. The cells were observed by Axio microscope (Zeiss, Goettingen, Germany). Images were analyzed by Zen-10 software (Zeiss, Goettingen, Germany).

### Proteomics—sample preparation, LC/MS, and data analysis

LGG was grown in TSB or TSB with 1% sugar for 24 h at 37 °C. For proteomics with metabolites, LGG was grown in TSB or TSB with 1% glucose or 20 µM acetic acid or 10 µM acetyl-CoA for 24 h in 37 °C. To process an equal number of bacteria, the OD_600_ of the cultures was compared. The cell pellets were subjected to in-solution tryptic digestion using the suspension trapping (S-trap) method as previously described^54^. Briefly, bacterial cell pellets were homogenized in the presence of lysis buffer containing 5% SDS in 50 mM Tris-HCl, pH 7.4. Lysates were incubated at 96°C for 5 min, followed by six cycles of 30 s of sonication (Bioruptor Pico, Diagenode, USA). Protein concentration was measured using a BCA assay (Thermo Scientific, USA). 50 ug of total protein was reduced with 5 mM dithiothreitol and alkylated with 10 mM iodoacetamide in the dark. Each sample was loaded onto S-trap microcolumns (Protifi, USA) according to the manufacturer’s instructions. After loading, samples were washed with 90:10% methanol/50 mM ammonium bicarbonate. Samples were then digested with trypsin (1:50 trypsin/protein) for 1.5 h at 47 °C. The digested peptides were eluted using 50 mM ammonium bicarbonate. Trypsin was added to this fraction and incubated overnight at 37 °C. Two more elutions were made using 0.2% formic acid and 0.2% formic acid in 50% acetonitrile. The three elutions were pooled together and vacuum-centrifuged to dry. Samples were resuspended in H_2_O with 0.1% formic acid and subjected to solid phase extraction (Oasis HLB, Waters, Milford, MA, USA) according to manufacturer instructions and vacuum-centrifuged to dryness. Samples were kept at −80 °C until further analysis.

#### Liquid chromatography

ULC/MS grade solvents were used for all chromatographic steps. Dry digested samples were dissolved in 97:3% H2O/acetonitrile + 0.1% formic acid. Each sample was loaded using split-less nano-Ultra Performance Liquid Chromatography (10 kpsi nanoAcquity; Waters, Milford, MA, USA). The mobile phase was: A) H2O + 0.1% formic acid and B) acetonitrile + 0.1% formic acid. Desalting of the samples was performed online using a reversed-phase Symmetry C18 trapping column (180 µm internal diameter, 20 mm length, 5 µm particle size; Waters). The peptides were then separated using a self-packed analytic column containing ReproSil-Pur 120 C18-AQ resin (100 µm internal diameter column, 250 mm length, 1.9 µm particle size; Dr. Maisch, Germany) at 0.35 µL/min. Peptides were eluted from the column into the mass spectrometer using the following gradient: 4% to 30%B in 155 min, 30% to 90%B in 5 min, maintained at 90% for 5 min and then back to initial conditions.

#### Mass Spectrometry

The nanoUPLC was coupled online through a nanoESI emitter (10 μm tip; New Objective; Woburn, MA, USA) to a quadrupole orbitrap mass spectrometer (Q Exactive HF, Thermo Scientific) using a FlexIon nanospray apparatus (Proxeon). Data were acquired in data dependent acquisition (DDA) mode, using a Top10 method. MS1 resolution was set to 120,000 (at 200 *m/z*), with a mass range of 375–1650 *m/z*, AGC of 3e6, and maximum injection time of 60 msec. MS2 resolution was set to 15,000, quadrupole isolation, 1.7 m/z, AGC of 1e5, dynamic exclusion of 50 sec, and maximum injection time of 60 msec.

#### Data processing and analysis

Raw data were processed with MaxQuant v1.6.6.0^55^. The data were searched with the Andromeda search engine against LGG protein sequences extracted from NCBI Reference Sequence NC_017482.1 and appended with common lab protein contaminants. Enzyme specificity was set to trypsin and up to two missed cleavages were allowed. Fixed modification was set to carbamidomethylation of cysteines and variable modifications were set to oxidation of methionines, and deamidation of glutamines and asparagines. Peptide precursor ions were searched with a maximum mass deviation of 4.5 ppm and fragment ions with a maximum mass deviation of 20 ppm. Peptide and protein identifications were filtered at an FDR of 1% using the decoy database strategy (MaxQuant’s “Revert” module). The minimal peptide length was 7 amino acids and the minimum Andromeda score for modified peptides was 40. Peptide identifications were propagated across samples using the match-between-runs option. Searches were performed with the label-free quantification option selected. The quantitative comparisons were calculated using Perseus v1.6.0.7. Decoy hits were filtered out and only proteins that had at least 2 valid values after logarithmic transformation in at least one experimental group were kept. Principle component analysis (PCA) of the log intensity values and heat maps were performed/generated in R. Student’s *t* tests followed by FDR correction, after logarithmic transformation, were used to identify significant differences between the experimental groups, across the biological replica. Fold changes were calculated based on the ratio of geometric means of the different experimental groups. GO-term annotation of NC_017482.1 proteins was performed with Blast2Go tool. GO-terms over representation analysis (ORA) was performed with the WebGestalt (web-based Gen Set Analysis Toolkit)^15^ on the upregulated set of proteins (FDR > 0.05, fold change >2) and using a custom database of the genome’s GO annotation performed with the Blast2Go tool. The cellular location for upregulated proteins (FDR > 0.05, fold change >2) was predicted using PSORT Server v. 3.0 at https://www.psort.org/psortb/^56^.

### Peptidoglycan purification, preparation of muropeptides, and LC-MS

Peptidoglycan (PG) sacculi were purified from planktonic cultures and biofilm colonies. Cells were collected by centrifugation (10,000 × *g*, 5 min) and kept at −20 °C. Frozen cells pellets were washed with 1 M NaCl, resuspended in 8% SDS in 0.1 M Tris/HCl pH 6.8, and boiled for 30 min. The suspension was then centrifuged (10,000 × *g*, 5 min) to collect the pellet, and washed five times with dH_2_O to remove SDS. Then the samples entered a sonifier water bath for 30 min and centrifuged. The pellet was then suspended in 15 µg/mL DNAse, 60 µg/mL RNAse in 0.1 M Tris/HCl pH 6.8 and incubated for 60 min at 37 °C, with gentle shaking. This was followed by treatment with 50 μg/mL and incubated at 37 °C for an additional 60 min, with gentle shaking. To inactivate the enzymes, the suspension was boiled for 3 min and then centrifuged (5 min at 10,000 rpm) and washed once with dH_2_O. Samples were then boiled again in 4% SDS (Sodium dodecyl sulfate) for 30 min, and washed five times with dH_2_O to remove SDS. The PG pellet was lyophilized, weighed, and stored at −20 until preparation for MS.

#### Preparation for MS

The pellet was resuspended in digestion buffer (12.5 mM sodium dihydrogen-phosphate, pH 5.5) with mutanolysin solution (5.000 U/mL) for 16 h at 37 °C with gentle shaking. To reduce muropeptides, equal volume of the solution of disaccharide peptides and of borate buffer (0.5 mM, pH 9.0) were mixed and incubated for 20 min at room temperature. pH was adjusted to <4 with (1:5) phosphoric acid and filtered through 0.22 µm.

#### HPLC-MS/MS

An UltiMate 3000 UHPLC+ focused LC-MS system (Thermo Scientific™) coupled with a Q Exactive™ Focus Hybrid Quadrupole-Orbitrap™ Mass Spectrometer (Thermo Scientific™) was used for LC/HRMS analysis. Muropeptides were separated using a C18 analytical column (Accucore TM C18, 2.6 µm particles, 100 × 2.1 mm; Thermo Fisher Scientific), column temperature at 50 °C. The flow rate was 0.2 mL/min when solvent A was 100% water with 0.1% formic acid, and solvent B was 100% acetonitrile, and 0.1% formic acid. 10 µl sample injected; MS/MS data were acquired over 40 min with a gradient of 0–12.5% B for 25 min, 12.5–20% B for 5 min, and held at 20% B for 5 min, and the column was re-equilibrated for 10 min under the initial conditions. The first 2 and last 5 min were excluded. The Q Exactive Focus was operated under positive ionization mode. The measurement was set to top 3 MS/MS fragmentations. The NCE was set with collision energy of 10,17.5,25. All MS spectra were analyzed by Xcalibur and freestyle software (Thermo Scientific™). PCA analysis was performed using MetaboAnalyst5.0^57^.

### Transmission electron microscopy (TEM)

A LGG biofilm colony was grown on solid medium (1.5% agar) containing 50% tryptic soy broth (TSB) with or without (1% w/v) D-(+) - glucose, for 7 days. Cells were fixed with 3% paraformaldehyde and 2% glutaraldehyde in 0.1 M cacodylate buffer containing 5 mM CaCl2 (pH 7.4), then post-fixed in 1% osmium tetroxide supplemented with 0.5% potassium hexacyanoferrate trihydrate and potassium dichromate in 0.1 M cacodylate (1 h), stained with 2% uranyl acetate in water (1 h), dehydrated in graded ethanol solutions and embedded in agar 100 epoxy resin (Agar scientific Ltd., Stansted, UK). Ultrathin sections (70–90 nm) were viewed and photographed with a FEI Tecnai SPIRIT (FEI, Eindhoven, Netherlands) transmission electron microscope operated at 120 kV and equipped with an EAGLE CCD Camera.

### Image analysis for cell wall thickness quantification

We quantified the mean and standard deviation of bacteria cell wall thickness from TEM images, by manually drawing the inner and outer cell wall borders, and then automatically matching the pairs of inner and outer boundaries to create a ring-like region of interest and quantifying the local thickness along the centerline (skeleton) of each cell wall. Manual drawing was done in Fiji^58^ and the boundaries were saved as regions of interest (ROIs) files. Automatic quantification was done using a Fiji macro that reads the TEM image together with the matching ROI file. Color-coded visualization of the results were created using the MorphoLibJ^59^ plugin. The macro is available at: https://github.com/WIS-MICC-CellObservatory/BacteriaCellWallThickness. All the images were manually calibrated using the scale bar, in order to get the measurements with proper calibration.

### Imaging flow cytometry

Biofilm colonies were cultured and incubated as mentioned in the first section. Colonies were harvested after 72 h and separated with mild sonication. For cell wall labeling, cells were gently centrifuged, resuspended in 100 µl of PBS supplemented with WGA-FITC (50 µg/mL, Invitrogen) or BODIPY™ FL vancomycin (10 µg/mL, Invitrogen), incubated for 15 min at room temperature, and washed twice with PBS before imaging. Data were acquired by ImageStreamX Mark II (AMNIS, Austin, Tx) using a 60× lens (NA = 0.9). The laser used was at 785 nm (5 mW) for side scatter measurement. During acquisition, bacterial cells were gated according to their area (in square microns) and side scatter, which excluded the calibration beads (that were run in the instrument along with the sample). For each sample, 100,000 events were collected. Data were analyzed using IDEAS 6.2 (AMNIS). Focused events were selected by the Gradient RMS, a measurement of image contrast. Cells stained with WGA-FITCH or BODIPY™ FL vancomycin were selected using the intensity (the sum of the background subtracted pixel values within the image) and max pixel values (the largest value of the background-subtracted pixels) of the green channel (Ch02). Cell wall intensity was quantified using the mean pixel feature (the mean of the background-subtracted pixels contained in the input mask).

### Scanning electron microscopy (SEM)

LGG biofilm colonies were grown on solid medium (1.5% agar) containing 50% tryptic soy broth (TSB) with or without (1% w/v) D-(+)-glucose, for 3 days. Then the biofilms were fixed overnight at 4 °C with 2% glutaraldehyde, 3% paraformaldehyde, 0.1 M sodium cacodylate (pH 7.4) and 5 mM CaCl2, dehydrated, and dried as described by Bucher et al.^21^. Mounted samples were coated with a 3 nm thick Ir layer (Safematick). The imaging by secondary electron (SE) detector was preformed using a high-resolution Carl Zeiss Ultra 55 or Sigma scanning electron microscope.

### Biofilm formation assay

For biofilm growth, 1 µL of LGG starter culture was diluted (1:100) in 200 µL 50% TSB, TSB supplemented with (1% w/v) D-(+)-glucose, (1% w/v) D-(+)-raffinose, (1% w/v) D- (+)- mannose or TSB supplemented with (1% w/v) D-(+) – glucose + buffer, in 96-well polystyrene plates and incubated for 72 h at 37 °C. For biofilm formation with metabolites, acetic acid, acetyl-coA, pyruvate, and lactic acid were added to TSB liquid medium in different concentrations. Biofilm formation was assessed by crystal violet staining. After 72 h, planktonic cells were removed by pipetting, and wells were washed with DDW (Deuterium-depleted water). The adherent cells were stained with 0.05% crystal violet stain for 30 min. The stain was then removed, and the wells were washed with DDW. 100% ethanol was added to the wells for 15 min. Crystal violet intensity was determined by a spectrophotometer (OD 575 nm). For calculating the ratio between planktonic cells and attached cells, 2 µL of LGG starter culture was diluted (1:100) in 2 mL of 50% TSB, TSB supplemented with (1% w/v) D-(+)-glucose, (1% w/v) D-(+)- raffinose, (1% w/v) D- (+)-mannose or TSB supplemented with (1% w/v) D-(+) – glucose + buffer in 12-well polystyrene plates and incubated for 24 h at 37 °C. The planktonic cells were removed and their OD_600_ measured. The adherent cells were resuspended in PBS (2 mL) and their OD_600_ measured. The ratio calculated was adherent cell OD_600_/planktonic cell OD_600_.

### Interaction assay

LGG, *S. typhimurium, E. faecalis, and P. aeruginosa* biofilm colonies were grown as mentioned in the first section. These bacteria were inoculated on solid medium (1.5% agar) containing 50% tryptic soy broth (TSB) with or without (1% w/v) D-(+) - glucose or D-(+)- raffinose next to each other at a distance of 0.2 mm for 7 days. As a control, each bacterium was inoculated separately. All images were taken with a Stereo Discovery V20” microscope (Tochigi, Japan) with objectives Plan Apo S ×0.5 FWD 134 mm or Apo S ×1.0 FWD 60 mm (Zeiss, Goettingen, Germany) attached to a high-resolution microscopy Axiocam camera. Data were generated and processed using Axiovision suite software (Zeiss).

### Conditioned media (CM) acquisition

LGG planktonic cultures, incubated for 24 h, at 37 °C were spun for 20 min at 4 °C at 4000 *g* to remove cells. The supernatant was than filtered through a 0.22 µm filter (Corning Incorporated, USA). Then the supernatant was separated by size to a large (>3 kDa) and small (<3 kDa) fraction using an Amicon ultrafiltration system with 3 kDa filter (Millipore, Ireland). The fraction was then filtered again through a 0.22 µm filter (Millipore).

### CFU assay

*S. typhimurium, E. faecalis, and P. aeruginosa* bacterial cultures were inoculated into a liquid medium of 50% TSB supplemented with (1% w/v) D-(+) - glucose with 30% conditioned media (CM), incubated for 24 h, without shaking, at 37 °C. Then, the samples were serially diluted x10 into 96 well plates and 20 µL from each sample was plated on solid LB agar (1.5 % agar) using a multichannel pipette with the dot-spot technique. CFU enumeration was carried out following overnight incubation at 37 °C.

### Proteinase K treatment

Large (>3 kDa) and small (<3 kDa) fractions of conditioned media (CM) from LGG grown with TSB supplemented with (1% w/v) D-(+) – glucose treated with 200 µg/ml proteinase K (proteinase K from *Tritirachium album*, Sigma) for 2 h in 37 °C with shaking. To remove the enzyme, the treated CM was filtered using an Amicon ultrafiltration system with 10 kDa filter (Millipore, Ireland). Then *S. typhimurium* and *E. faecalis* cultured cells grown overnight were diluted 1:100 in 200 μL liquid medium containing 30% treated conditioned medium and non-treated conditioned medium of large fraction (>3 kDa) and small fraction (<3 kDa), respectively. Cells were grown with agitation at 37 °C for 20 h in a microplate reader (Thremo Fisher), and the optical density at 600 nm (OD_600_) was measured every 30 min.

### LC-MS for polar metabolite analysis

LGG planktonic cultures grown in 50% TSB, TSB supplemented with (1% w/v) D-(+) - glucose, (1% w/v) D-(+)- raffinose or (1% w/v) D- (+)- mannose for 24 h at 37 °C. To get an equal number of bacteria the OD_600_ of the bacteria was compared, and after that washed twice with PBS. For polar metabolite analysis in the polar phase samples, the lyophilized pellets were dissolved using 100 µL DDW-methanol (1:1), centrifuged twice (at maximum speed) to remove possible precipitants, and were injected into LC-MS system. Polar analysis in the polar phase was done as following: Analysis was performed using Waters Acquity I class UPLC System combined with a mass spectrometer (Thermo Exactive Plus Orbitrap) operated in a negative ionization mode. The LC separation was done using the SeQuant Zic-pHilic (150 mm × 2.1 mm) with the SeQuant guard column (20 mm × 2.1 mm) (Merck). The Mobile phase B: acetonitrile and Mobile phase A: 20 mM ammonium carbonate with 0.1% ammonia hydroxide in water: acetonitrile (80:20, v/v). The flow rate was kept at 200 μL min^−1^ and gradient as follows: 0–2 min 75% of B, 17 min 12.5% of B, 17.1 min 25% of B, 19 min 25% of B, 19.1 min 75% of B, 23 min 75% of B.

#### Polar metabolites data analysis

The data processing was done using TraceFinder Thermo Fisher software. Polar metabolites were identified by accurate mass, retention time, and isotope pattern, and verified using an in-house mass spectra library.

### Volatiles collection and analysis

The samples were prepared for analysis in 20 mL headspace vials. The headspace (300 mL) above cultures was actively sampled. Volatile compound (VC) analysis was conducted on a thermal desorption-gas chromatography time-of-flight mass spectrometer (GC-TOF-MS) platform (Leco BT, Germany) combined with a Gerstel MPS autosampler (Germany). The VOCs (volatile organic compounds) were collected using SPME PDMS/DVB (pink) fiber at 30 °C for 15 min and desorbed for 3 min using temperature 220 °C. The GC column (ZB-624PLUS column, 30 m, 0.32 mm internal diameter, 1.8 μm film thickness, Phenomenex) was held at an initial temperature of 40 °C for 3 min, ramped to 205 °C at 7 °C min and held at 205 °C for 1 min. The GC runtime was 28 min. The TOF-MS was in electron ionization mode set at 70 eV. The source temperature was set to 250 °C, and spectra were acquired in dynamic range extension mode at 10 scans s−1 over a range of 39–500 *m/z*.

#### GC data processing

GC-TOF-MS data were acquired and analyzed using ChromaTof (Leco, Germany). Chromatographic peaks and mass spectra were cross-referenced with National Institute of Standards and Technology (NIST 17) and Wiley libraries for putative identification purposes (matching factor >750 match).

### Statistical analysis

Statistical analyses were performed with GraphPad Prism 9.0 (GraphPad 234 Software, Inc., San Diego, CA) unless explicitly stated otherwise. Relevant statistical tests are mentioned in the indicated legends of the figures.

### Reporting summary

Further information on research design is available in the Nature Research Reporting Summary linked to this article.

### Supplementary information

Supporting Information

Supplementary Table 1

Supplementary table 2

Supplementary Table 3

Supplementary Table 4

Reporting Summary

## Data Availability

All required data for the main and supporting figures are provided with the manuscript.
