# Supplementary material for: Metabolic inputs in the probiotic bacterium Lacticaseibacillus rhamnosus contribute to cell-wall remodeling and increased fitness
Source: NPJ Biofilms Microbiomes. 2023 Sep 26;9:71. doi: 10.1038/s41522-023-00431-2 (PMC10522624; doi:10.1038/s41522-023-00431-2)
Supplement: Supplementary file 1 — Supporting Information [file 41522_2023_431_MOESM1_ESM.pdf]

Metabolic inputs in the probiotic bacterium *Lacticaseibacillus rhamnosus* GG and their contribution to cell wall remodeling, biofilm formation and antimicrobials production

Ronit Suissa[1], Tsviya Olender[2], Sergey Malitsky[3], Ofra Golani[3], Sondra Turjeman[4], Omry Koren[4*], Michael M. Meijler[1*] and Ilana Kolodkin-Gal[2,5*]

[1] Department of Chemistry, Ben-Gurion University of the Negev, Be'er Sheva, Israel

[2] Department of Plant Pathology and Microbiology, Faculty of Agriculture, Food and Environment, The Hebrew University of Jerusalem, Rehovot, Israel.

[3] Life Science Core Facilities, Weizmann Institute of Science, Rehovot, Israel

[4] Azrieli Faculty of Medicine, Bar-Ilan University, Safed, Israel.

[5] Current address: The Scojen institute for Synthetic Biology, Reichman University Herzliya, Israel.

Correspondence: Omry.Koren@biu.ac.il; meijler@bgu.ac.il, ilana.kolodkin@runi.ac.il

Supplementary Figures S1-S14

Supplementary Methods

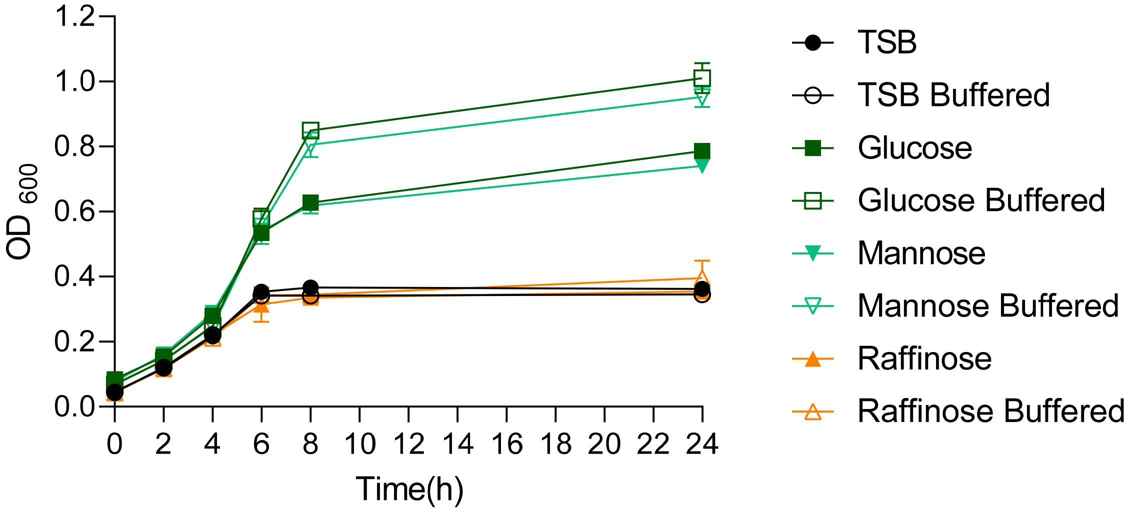

Supplementary Figure 1. Growth curve of LGG grown in TSB, TSB supplemented with glucose (1% W/V), mannose (1% W/V), raffinose (1% W/V) or TSB medium supplemented with different sugar (1% W/V) + buffer. LGG cultured cells grown for overnight were diluted 1:100 in 10ml liquid medium and the optical density at OD_{600} was measured at 0h,2h,4h,6h,8h and 24h.

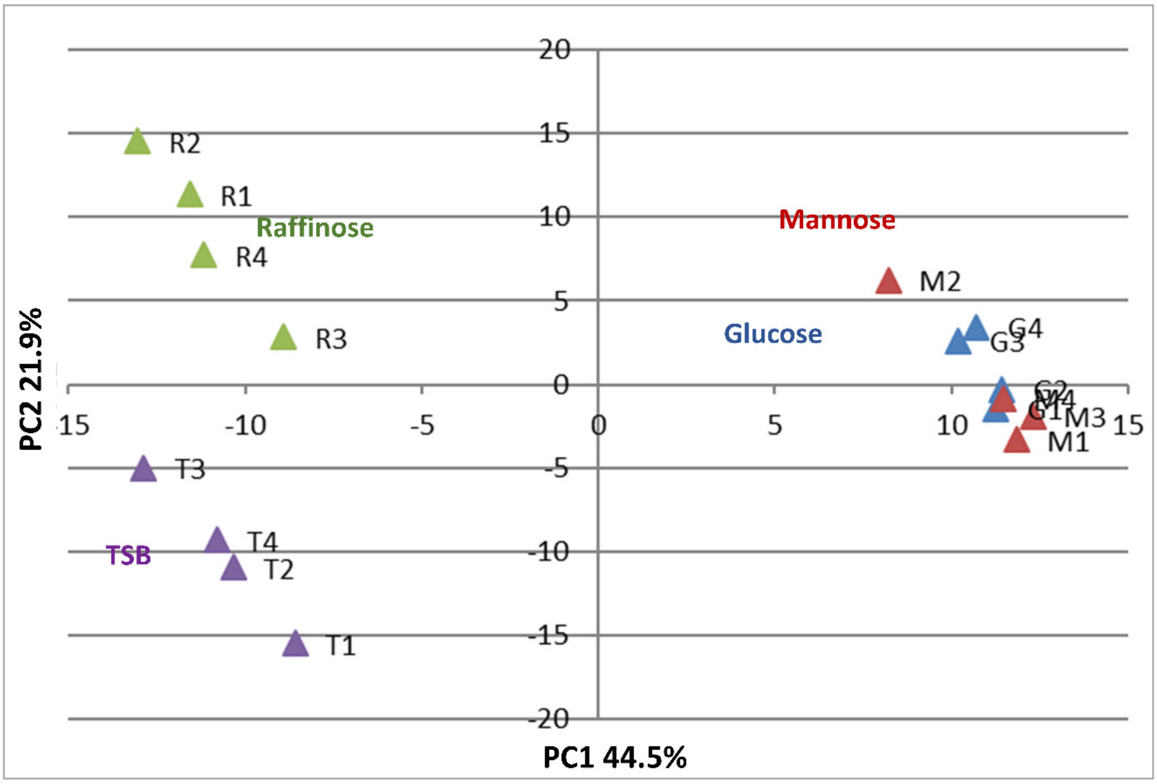

Supplementary Figure 2. Principal component analysis plot of metabolomics analysis from LGG grown in liquid TSB medium or TSB medium supplemented with glucose (1% W/V), mannose (1% W/V) or raffinose (1% W/V). Raffinose [R1-R4], Glucose [G1-G4], Mannose [M1-M4], Non-supplemented TSB [T1-T4]

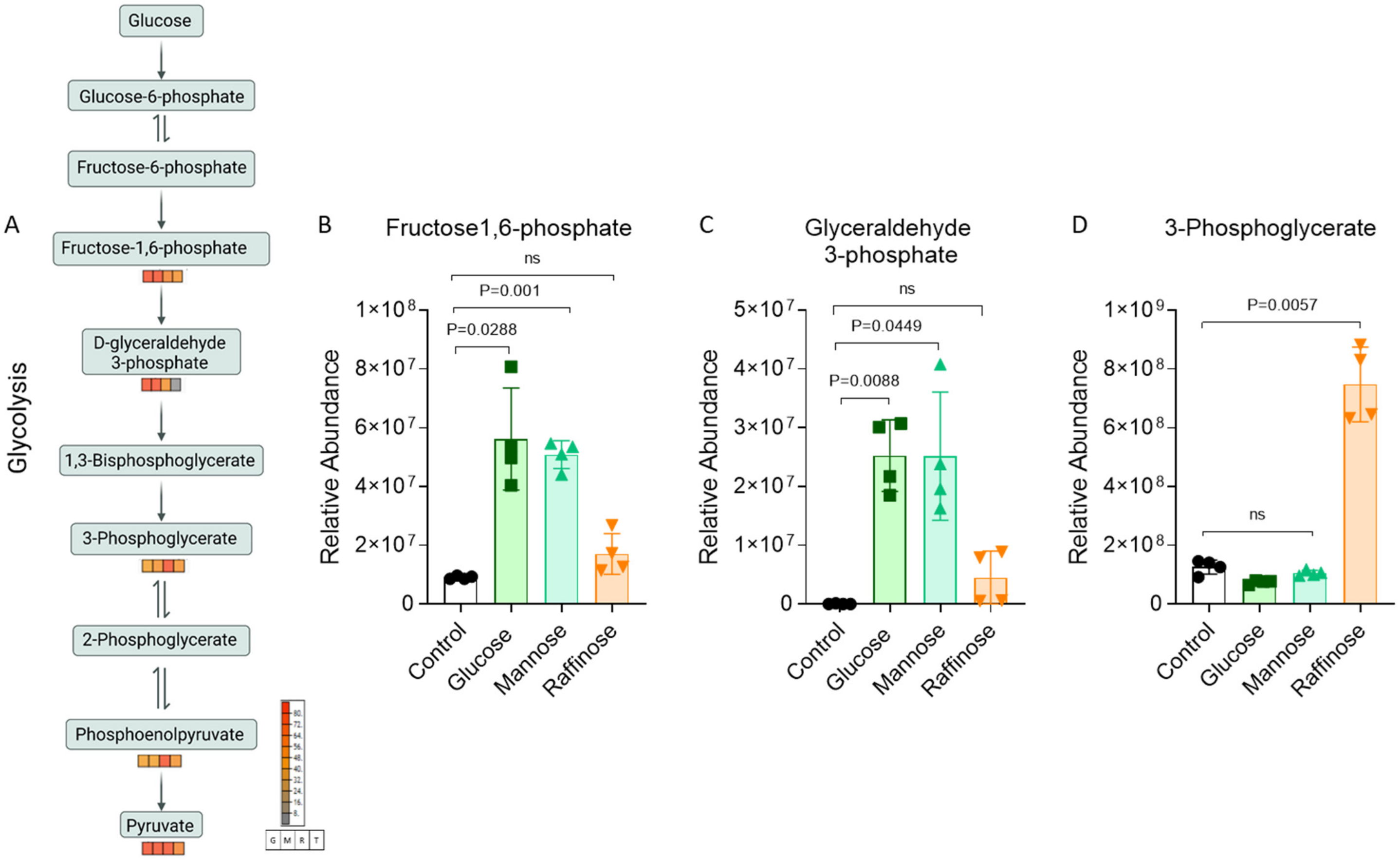

Supplementary Figure 3. (A) MetaCyc metabolic pathway of glycolysis based on metabolomics analysis from LGG grown in TBS medium or TSB medium with glucose (1% W/V), mannose (1% W/V) or raffinose (1% W/V). Relative abundance of **(B)** Fructose 1'6-phosphate **(B)** Glyceraldehyde 3-phosphate (**D)** 3-phosphoglycerate. The images represent 4 independent repetitions. Statistical analysis was performed using Brown-Forsythe and Welch's ANOVA with Dunnett's T3 multiple comparisons test. $p < 0.05$ was considered statistically significant.

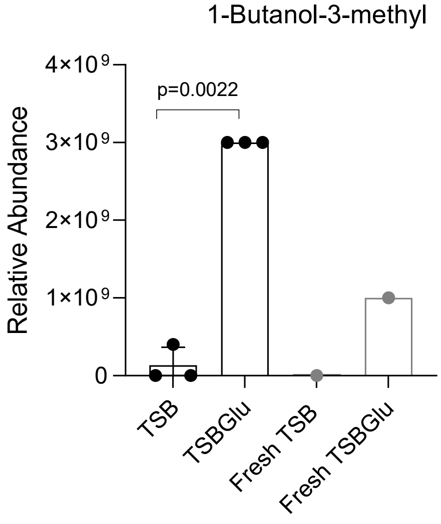

Supplementary Figure 4. Relative abundance of 1 Butanol, 3-methyl identified in TBS medium or TSB medium with glucose (1% W/V). Gray bars represent the background (fresh medium TSB or TSB with glucose (1% W/V).

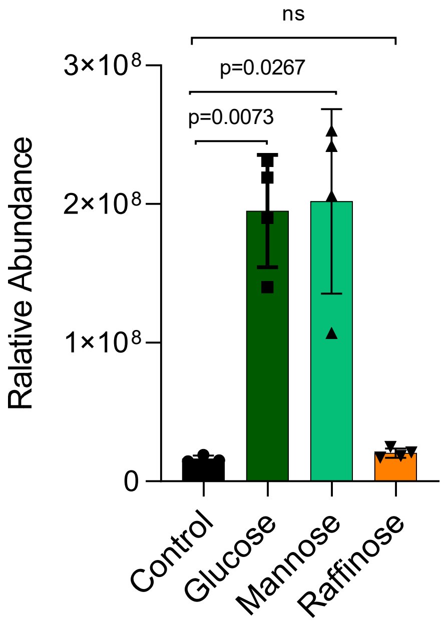

Supplementary Figure 5. Protein relative abundance of Class A sortase between samples. Statistical analysis was performed using Brown-Forsythe and Welch's ANOVA with Dunnett's T3 multiple comparisons test. $p < 0.05$ was considered statistically significant.

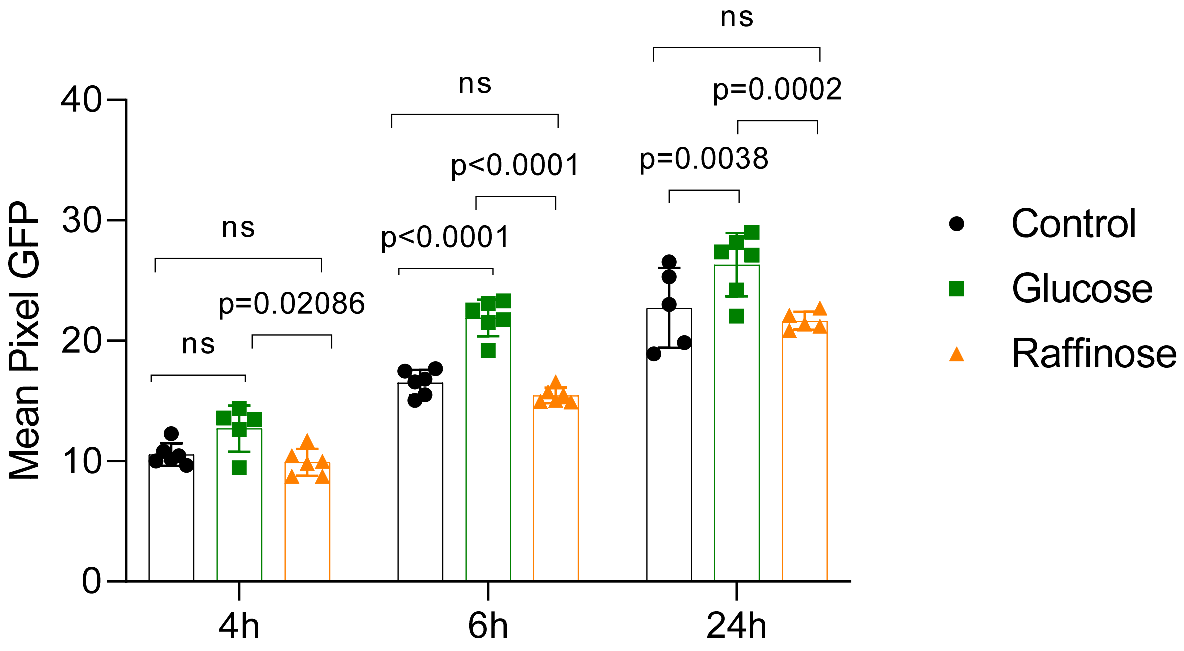

Supplementary Figure 6. Imaging Flow cytometry analysis of the mean pixel intensity of LGG grown in TSB (control), TSB supplemented with glucose (1% W/V) or raffinose (1% W/V). Data were collected after 4h,6h and 24h and 50,000 cells were counted. Cells were labeled using BODIPY™ FL Vancomycin. Graphs represent mean ± SD from 2 independent experiments (n = 6). Statistical analysis was performed using two-way ANOVA with Tukey's multiple comparisons test. $p < 0.05$ was considered statistically significant.

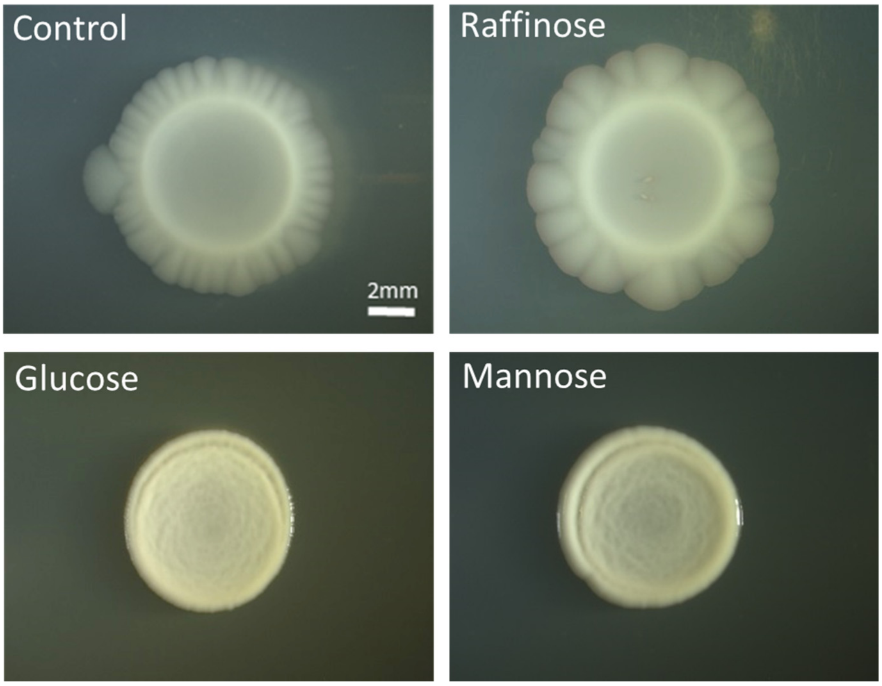

Supplementary Figure 7. LGG grown on solid TSB (control), TSB supplemented with glucose (1% W/V), mannose (1% W/V) or raffinose (1% W/V). Biofilms were grown at 37° C in CO_2 enriched environment for 7 days.

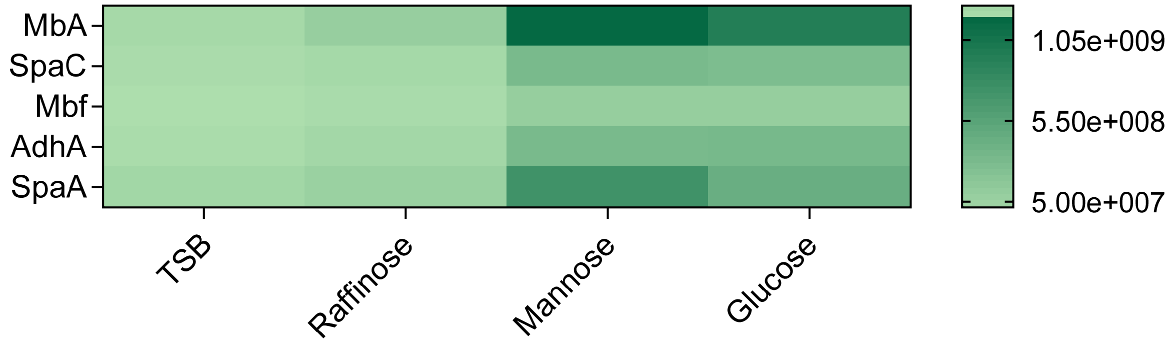

Supplementary Figure 8. Heat map based on fold differences in the intensity of adhesion proteins identified in proteomics analysis.

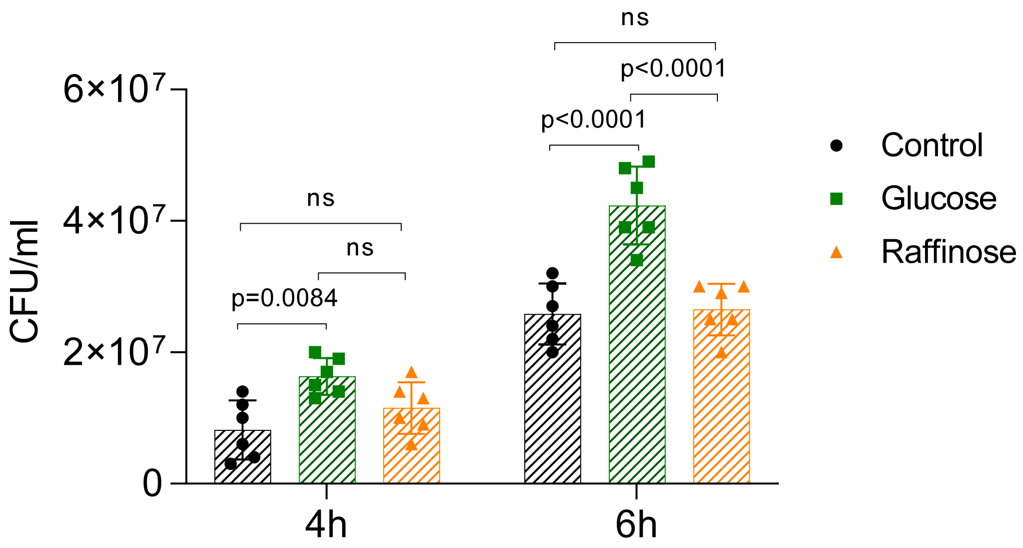

Supplementary Figure 9. Adherence of LGG grown in TSB (control), TSB supplemented with glucose (1% W/V) or raffinose (1% W/V) to porcine mucin in a microtiter plate. Adherence to porcine mucin tested after 4h and 6h growth. Y-axis represents the number of bacteria adherent to mucin by CFU/ml, graphs represent mean ± SD from 2 independent experiments (n = 6). Statistical analysis was performed using two-way ANOVA with Tukey's multiple comparisons test. $p < 0.05$ was considered statistically significant.

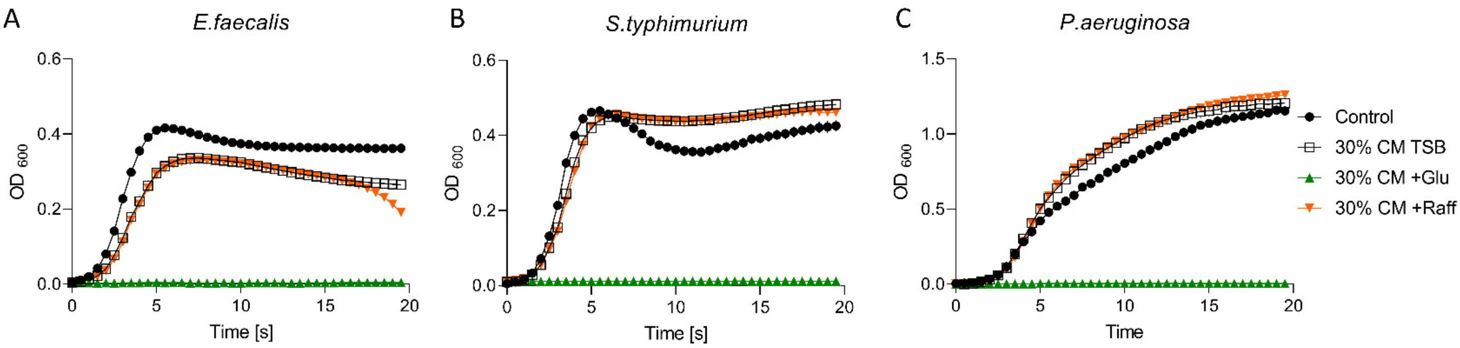

Supplementary Figure 10. Growth curves of **(A)** *E. faecalis* (**B)** *S. typhimurium* and **(C)** *P. aeruginosa* in 96 well plates in 37°C with shaking in TSB with glucose. Cells were supplemented with 30% conditioned medium (CM) derived from LGG grown in liquid TSB medium or TSB medium supplemented with glucose (1% W/V) or raffinose (1% W/V).

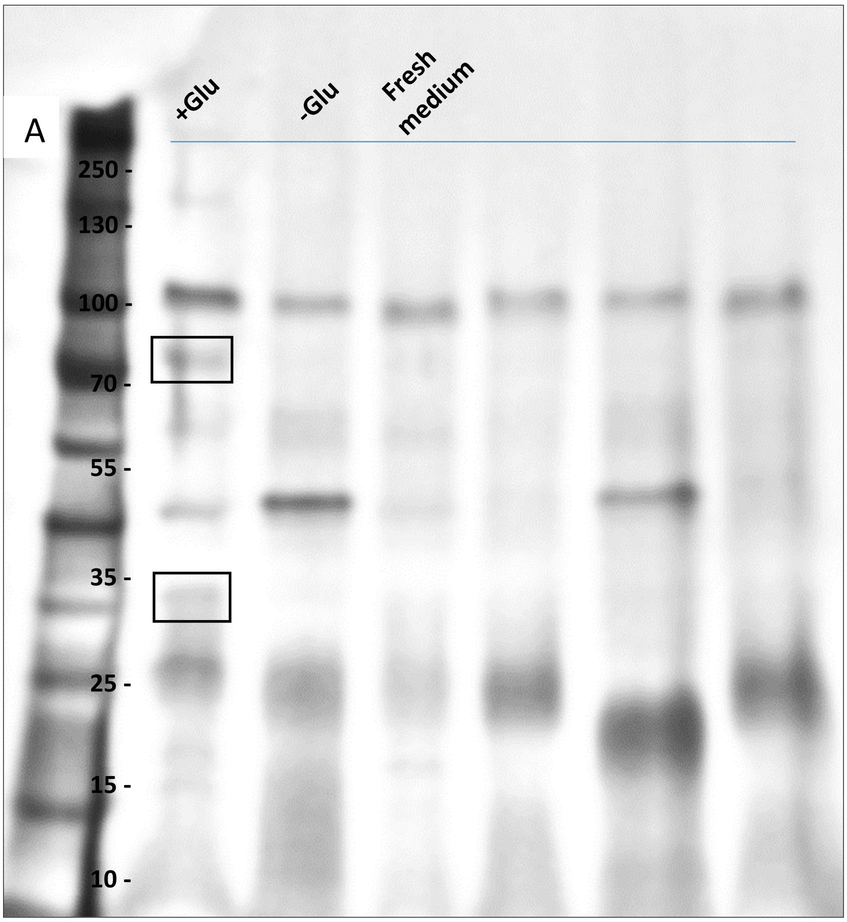

B

1 MVDSKKVLSV TAGFVGAAGL AALATGANTV SASTGTVSYK **SGATTVWNSP SWHQVKRYVT**

61 **FGDTVQLLGK TVDQNGATWY KVGDNQWIPE LYLNVAGK**TA TVETPSSAAS QTAVSQA

121 QAPTSQAPAT QTPAAPQTDT QTANTQLYVK NIGSAVTVWT TPAYTHATGQ YLEGSQTLTA

181 VAQQQANGET WYR**LANGGYV PAR**FVSTTPV AVTPQPAAPQ SNEASVASTN TNAANDSAAA

241 SSAAASQAAA SSAAASTAAA NAAVASANAT ASQAAASEAA ASQAAASQAA ASQAAASQAA

301 ASQAAASQAA ASQAAASQAA ASQAAASQAA ANAAQQAPAN QANVTTTQVN ANQAQQQTAT

361 ATPAVNTSNQ TAAVSASRQA K**IQAVIAIAE QQVGKPYVWG GK**GPNSFDCS GLMYYAFLNG

421 AGVNIGGWTV PQESSGTQVS LSALQPGDLL FWGSHGSTYH VALYIGGGTM IQAPQPGENV

481 K**YTALAYFMP DFAVRPSL**

Supplementary Figure 11. (A)SDS-PAGE of CM >3 kDa from LGG grown in TSB medium or TSB supplemented with glucose (1% W/V) and fresh TSB as a background. **(B)** Coverage of the identification of Msp1/P75 in the big band. All gels were processed in parallel and derive from the same experiments

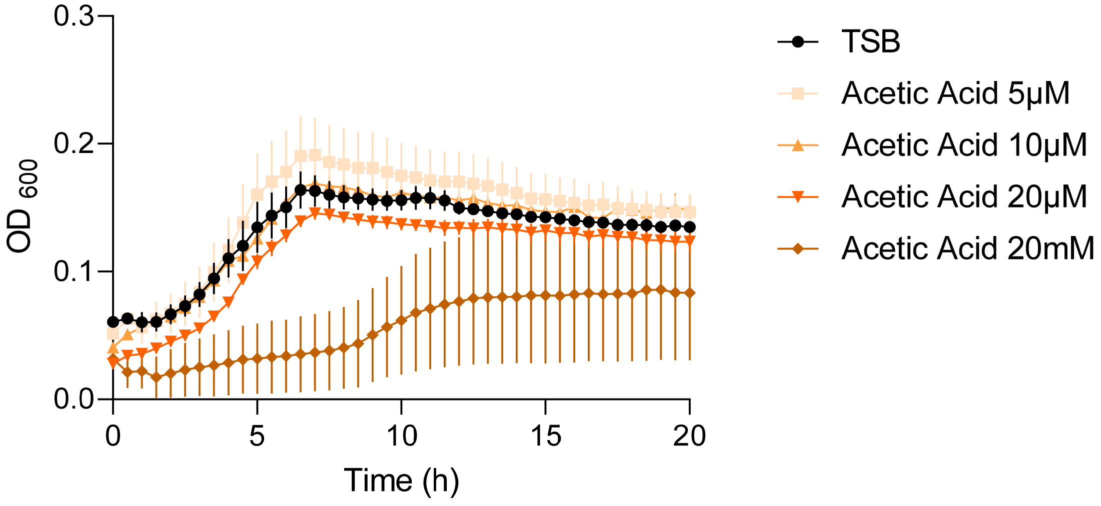

Supplementary Figure 12. Growth curves of LGG in 96 well plates in 37°C with shaking in TSB. Cells were supplemented with different concentrations of acetic acid.

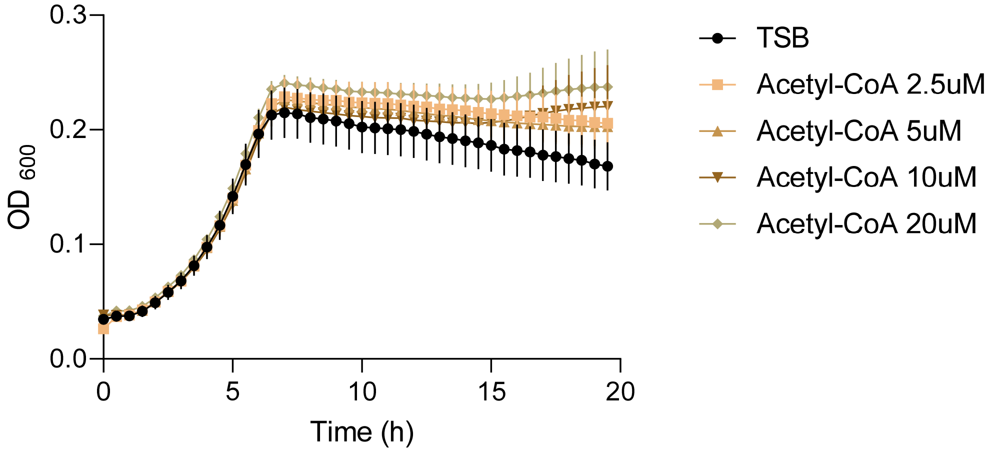

Supplementary Figure 13. Growth curves of LGG in 96 well plates in 37°C with shaking in TSB. Cells were supplemented with different concentrations of acetyl-coA.

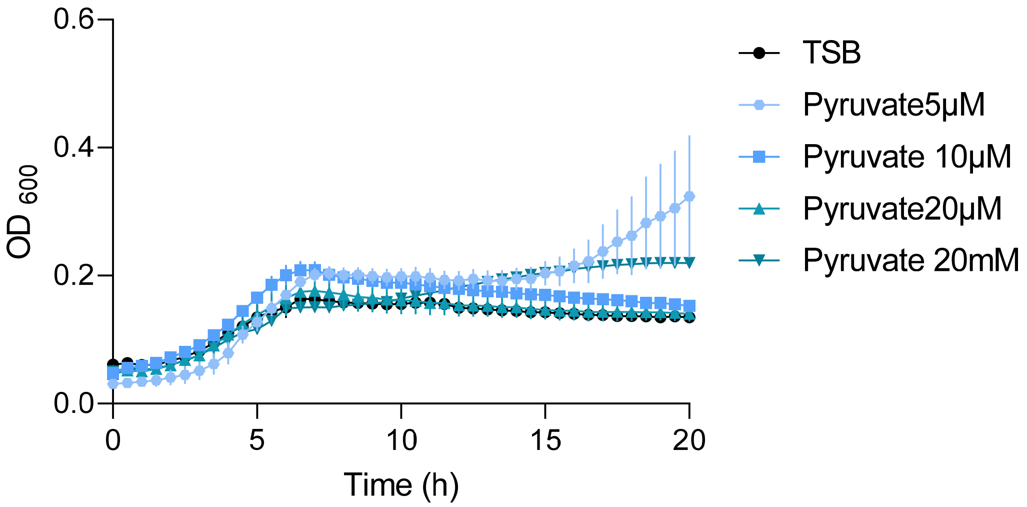

Supplementary Figure 14. Growth curves of LGG in 96 well plates in 37°C with shaking in TSB. Cells were supplemented with different concentrations of pyruvate.

Supplementary Methods

In gel proteolysis and mass spectrometry analysis

The big fraction driven from Lacticaseibacillus rhamnosus GG grown with or without glucose and fresh 25% TSB+1% glucose as a control were mixed with 5x SDS sample buffer (10% SDS, 250 mM Tris-HCl pH 6.8, 0.5 M DTT, 0.1% bromophenol blue, 50% glycerol). Lysis and denaturation were carried out by incubation at 95 °C for 5 min. Proteins from the resulting cell extracts were loaded on a 10% SDS-PAGE gel, separated in SDS-running buffer (25 mM Tris, 192 mM glycine, 0.1% SDS) for 45 min with 80-120 V with a system from Bio-Rad. Proteins were stained with the Silver Stain. Silver-stained gel was disdained with 30mM potassium hexacyanoferrate and 100mM sodium thiosulfate. The proteins in the gel were reduced with 2.8mM DTT (60ºC for 30 min), modified with 8.8mM iodoacetamide in 100mM ammonium bicarbonate (in the dark, room temperature for 30 min) and digested in 10% Acetonitrile and 10mM ammonium bicarbonate with modified trypsin (Promega) overnight at 37oC.

The resulting tryptic peptides were desalted using C18 tips (Homemade stage tips) dried and re-suspended in 0.1% Formic acid. The resulting tryptic peptides were resolved by reverse-phase chromatography on 0.075 X 200-mm fused silica capillary (J&W) packed with Reprosil reversed phase material (Dr Maisch GmbH, Germany). The peptides were eluted with linear 30 minutes gradient of 5% to 28% acetonitrile with 0.1% formic acid in water ,15 minutes gradient of 28% to 95% acetonitrile with 0.1% formic acid in water and 15 minutes at 95% acetonitrile with 0.1% formic acid in water at flow rates of 0.15 μl/min. Mass spectrometry was performed by a Q-Exactive plus mass spectrometer (Thermo) in a positive mode using repetitively full MS scan followed by High energy Collision Dissociation (HCD) of the 10 most dominant ion selected from the first MS scan. The mass spectrometry data was analyzed using Proteome Discoverer 1.4 software Using Sequest (Thermo) algorithm searching against the human proteome from the Uniprot database, and L. rhamnosus GG from the NCBI-nr database. Semi quantitation was done by calculating the peak area of each peptide based its extracted ion currents (XICs). The area of the protein is the average of the three most intense peptides from each protein. Results were filtered with 1% false discovery rate.

Imaging Flow Cytometry

LGG bacterial cultures were inoculated (1:100) into 10ml liquid medium TSB (control), TSB supplemented with glucose (1% W/V) or raffinose (1% W/V), incubated for 24h, no shaking, at 37°C. Cells were harvested after 4h,6h and 24h hours, washed once with PBS and separated with mild sonication. Cells were centrifuged, resuspended in 100 µl of PBS supplemented with BODIPY™ FL Vancomycin (10 µg/mL, Invitrogen), incubated for 15 min at room temperature, and washed twice with PBS before imaging. Data were acquired by ImageStreamX Mark II (AMNIS, Austin, Tx) using a 60× lens (NA=0.9). The laser used was at 785 nm (5 mW) for side scatter measurement. During acquisition, bacterial cells were gated according to their area (in square microns) and side scatter, which excluded the calibration beads (that run in the instrument along with the sample). For each sample, 100,000 events were collected. Data were analyzed using IDEAS 6.2 (AMNIS). Focused events were selected by the Gradient RMS, a measurement of image contrast. Cells stained with BODIPY™ FL Vancomycin were selected using the Intensity (the sum of the background subtracted pixel values within the image) and Max Pixel values (the largest value of the background-subtracted pixels) of the green channel (Ch02). Cell wall intensity was quantified using the Mean Pixel feature (the mean of the background-subtracted pixels contained in the input mask).

Mucin adhesion assay

Strains were assayed for adhesion to mucin in 96-well microtiter plates under sterile condition. Plates were coated with 100 µL of 10mg/ml porcine Mucin Type || (Sigma-Aldrich) in sterile Dulbecco's phosphate-buffered saline (PBS) at 4°C overnight. Wells were washed twice with sterile PBS to remove unbound mucin. LGG bacterial cultures were inoculated (1:100) into 10ml liquid medium TSB (control), TSB supplemented with glucose (1% W/V) or raffinose (1% W/V), incubated for 24h, no shaking, at 37°C. Cells were harvested after 4h and 6h hours, (7000 g for 2 min at 4°C) and the bacterial cells were resuspended in sterile PBS and adjusted to the optical density (OD_{600}) of 0.5. 100 µL of each strain was added to respective wells and allowed to adhere mucin for 2h at 37°C. Un-adhered bacterial cells were then withdrawn, and wells were

washed 3 times with 100 µL sterile PBS each. Adhered cells were released by treatment with 100 µL 0.1% (v/v) Triton X-100 in sterile PBS for 30 min at 37°C. The released bacterial cells were plated after appropriate dilution on MRS agar, and enumeration was carried out following 48-h incubation at 37°C.
